# Knockdown of a laccase in *Populus deltoides* confers altered cell wall chemistry and increased sugar release

**DOI:** 10.1111/pbi.12560

**Published:** 2016-04-15

**Authors:** Anthony C. Bryan, Sara Jawdy, Lee Gunter, Erica Gjersing, Robert Sykes, Maud A. W. Hinchee, Kimberly A. Winkeler, Cassandra M. Collins, Nancy Engle, Timothy J. Tschaplinski, Xiaohan Yang, Gerald A. Tuskan, Wellington Muchero, Jin‐Gui Chen

**Affiliations:** ^1^ BioEnergy Science Center and Biosciences DivisionOak Ridge National LaboratoryOak RidgeTNUSA; ^2^ The Biosciences CenterNational Renewable Energy LaboratoryGoldenCOUSA; ^3^ ArborGen IncRidgevilleSCUSA

**Keywords:** *Populus*, biofuel, cell wall, xylose, lignin, recalcitrance

## Abstract

Plant laccases are thought to function in the oxidation of monolignols which leads to higher order lignin formation. Only a hand‐full of laccases in plants have been functionally evaluated, and as such little is known about the breadth of their impact on cell wall chemistry or structure. Here, we describe a previously uncharacterized laccase from *Populus*, encoded by locus Potri.008G064000, whose reduced expression resulted in transgenic *Populus* trees with changes in syringyl/guaiacyl ratios as well as altered sugar release phenotypes. These phenotypes are consistent with plant biomass exhibiting reduced recalcitrance. Interestingly, the transgene effect on recalcitrance is dependent on a mild pretreatment prior to chemical extraction of sugars. Metabolite profiling suggests the transgene modulates phenolics that are associated with the cell wall structure. We propose that this particular laccase has a range of functions related to oxidation of phenolics and conjugation of flavonoids that interact with lignin in the cell wall.

## Introduction

The ability to break down plant cell walls is a key factor limiting the extraction of sugars that can be utilized for the production of ethanol during biomass fermentation for biofuel production. Plant cell wall resistance to digestion, that is recalcitrance, is governed by the inherent chemical composition and interpolymer interactions of the cell wall consisting of lignin, cellulose and hemicellulose. Cell wall composition and interaction of key polymers during biomass fermentation have been previously reviewed (Carpita, 2012; Loque *et al*., 2015). Currently, expensive pretreatments using high temperatures, harsh corrosives and enzymes are utilized to increase the extractability of sugars stored in cell walls (Blanch, 2012; Chundawat *et al*., 2011). Finding ways to alter cell wall composition or structure and reduce the severity of pretreatments is a key goal in developing cost‐effective biomass feedstocks for biofuel and bioproduct production. The ability to genetically modify biomass feedstocks can have a direct impact on the ability to extract sugars and therefore yield of transportation fuels from plant biomass. Identifying genes that regulate cell wall biosynthesis and composition and reduce recalcitrance is a critical step for efficient production of products from lignocellulosic biomass.

One major component of secondary cell walls that directly affects recalcitrance and prevents easy extraction of sugars is lignin (Chen and Dixon, 2007). Lignin is a polyphenolic heteromorphic polymer found in cell walls that adds structural support for cell walls and is created through the polymerization of the monolignols coniferyl alcohol, *p*‐coumaryl alcohol and sinapyl alcohol that form the guaiacyl (G), hydroxyl‐coumaryl (H) and syringyl (S) subunits, respectively. The formation of lignin polymers and the regulation of which subunits are utilized in lignin molecules is, however, still not well understood. It is known that increased quantity of lignin in cell walls reduces the saccharification of the cell walls and limits the access to primary carbohydrates, that is cellulose and hemicellulose (Chen and Dixon, 2007). Additionally, lignin subunit composition can also influence ruminant digestibility (Akin *et al*., 1986). Cell walls from different plant species, and even from different tissues from the same plant, can vary in their monolignol subunit composition. For example, gymnosperms typically have abundant G and low H subunits, while dicots contain mostly G and S subunits (Weng and Chapple, 2010). Syringyl to guaiacyl (S/G) ratio, in particular, is an important quality with a higher presence of S subunits making sugars more amenable to extraction (Studer *et al*., 2011). In addition, interaction of sugars with lignin has been shown to affect digestibility of biomass. Higher hemicellulose levels were shown to increase saccharification likely by interlinking with lignin to reduce lignocellulosic crystallinity, as were shown in *Miscanthus* (Si *et al*., 2015; Xu *et al*., 2012). While utilizing biomass with properties amenable to deconstruction and access to sugars, targeting genes to modulate the properties of lignin and its associations has been difficult due to the lack of understanding how plant cells regulate lignin polymerization and interpolymer interfaces.

Recent literature has provided new understanding of cell wall interactions and mechanisms regulating composition that may be manipulated for a desired product. Modifying or regulating linkages of lignin with phenolics has been shown to greatly affect biomass digestibility (Li *et al*., 2014; Wilkerson *et al*., 2014). On the other hand, high‐level lignin has been shown to be a positive factor on biomass saccharification in rice mutants (Li *et al*., 2015; Wu *et al*., 2013) and artificial cellulose–lignin interactions affect digestibility (Zhang *et al*., 2016), indicating the level of complexity of cell wall interactions and mechanisms. Properties of the cell wall, including composition, intermolecular interactions and interlinking, cellulose crystallinity and even the release of toxic compounds during pretreatment are all factors that affect accessibility and utilization of sugars for biofuel production.

Laccases are copper‐containing glycoproteins found in a wide range of organisms (Baldrian, 2006; Claus, 2003; Dittmer and Kanost, 2010; Dittmer *et al*., 2004; McCaig *et al*., 2005). Although they share significant homology, laccases appear to have functionally diverged within and between phylogenetic clades (Dittmer *et al*., 2004). Bacterial, fungal and insect laccases have been shown to function in the degradation of lignin, whereas higher plant laccases are thought to function in the polymerization of lignin subunits (Sharma and Kuhad, 2008). Additionally, even though laccases retained similar protein domains, molecular modelling suggests differences in protein folding and affinity for interacting with lignin, which may result in divergence of activity in lignin synthesis and degradation (Awasthi *et al*., 2015). Laccases are known to function in oxidation reactions involving various inorganic and organic substrates including phenolics and aromatic amines in plants. Studies in *Populus* and *Arabidopsis* suggest that laccases act not only in the biosynthesis of lignin but also may contribute to additional roles of cell wall chemistry or integrity (Ranocha *et al*., 2002; Ranocha *et al*., 1999; Zhao *et al*., 2013). In plants, it was thought that laccases may be involved in lignin biosynthesis based on their capability to oxidize lignin precursors and their localization in lignifying tissues (Bao *et al*., 1993; Driouich *et al*., 1992; Ranocha *et al*., 1999; Sterjiades *et al*., 1992). For example, over‐expression of the cotton laccase, *GaLACCASE 1 (LAC1)*, in *Populus* leads to increased lignin content with transgenic plants showing a 2.1%–19.6% increase in total lignin, indicating that laccases are involved in lignin biosynthesis (Wang *et al*., 2008). In *Arabidopsis*, insertional mutations in three laccase‐encoding genes completely abolished lignin accumulation (Zhao *et al*., 2013). Interestingly, the three laccases, *At*LAC4, 11 and 17, are not paralogous and show homology to different subfamilies of the laccase gene family, suggesting that lignin biosynthesis is not controlled by a single subfamily. A study in *Populus* indicated that transgenic trees, in which expression of the laccase gene *Pt*LAC3 was reduced, showed a threefold increase in phenolic content which accumulated in xylem ray parenchyma cells (Ranocha *et al*., 2002). In addition, xylem fibre cell walls were dramatically altered leading to severe deformation, indicating a defect in cell wall integrity and supporting the importance of this laccase in normal xylem cell wall structure and integrity (Ranocha *et al*., 2002). However, there was no significant change in lignin quantity or composition. Most laccases, especially in *Populus*, have yet to be studied, leaving numerous questions unanswered regarding the specific role individual laccases play in lignin biosynthesis or cell wall development.

To address these questions, in part, we created transgenic *Populus* lines with reduced expression of the *Populus LAC2*, encoded by locus Poptri.008G064000 (*PtLAC2*). This laccase is paralogous to the previously studied *PtLAC3* that showed significant effects on cell wall integrity. We reduced gene expression using gene‐specific RNAi to *PtLAC2* and measured lignin composition and total sugar release. We also performed GC‐MS metabolite profiling to assess changes in cell wall precursors. Results from our studies suggest that LAC2 likely is involved in reactions leading to structural changes in cell wall integrity. Down‐regulation of *LAC2* results in a disrupted cell wall assembly phenotype and other pleiotropic consequences, as described below, permitting a pretreatment‐dependent increase in release of glucose and xylose.

## Results

### Phylogenetic analysis of *Populus* LAC2

**Figure 1:**
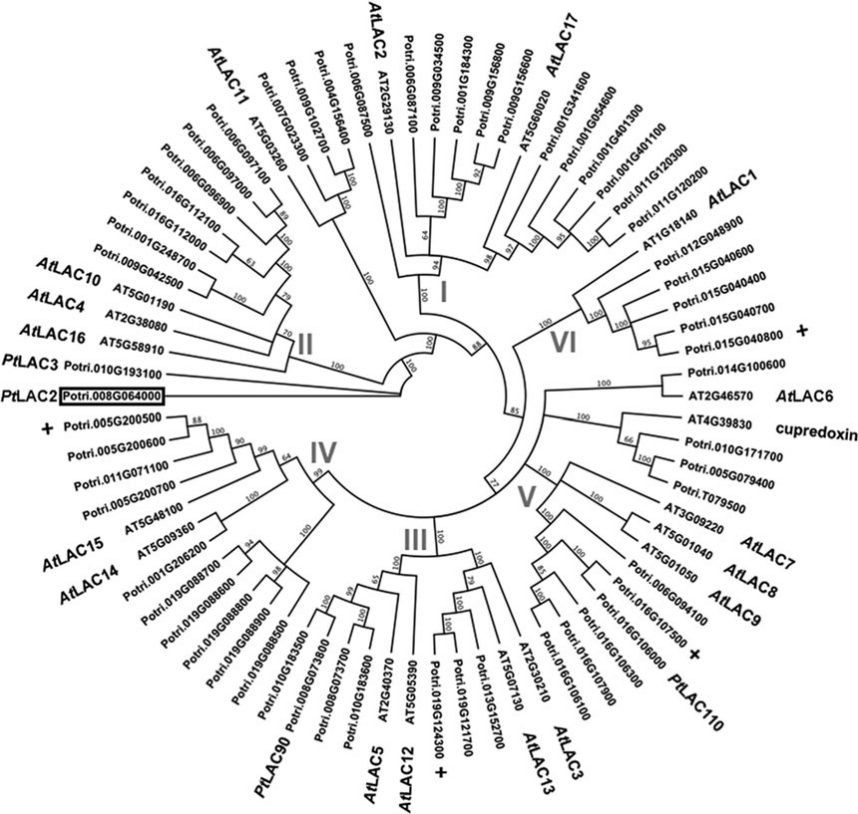
Phylogenetic analysis of Laccase *(LAC*) genes from *Populus trichocarpa* and *Arabidopsis thaliana*. The six subfamilies, indicated by Roman numerals, were previously described by McCaig *et al*. (2005) and *Arabidopsis LAC* genes named accordingly. *Populus trichocarpa LAC* genes were identified through BLAST from Phytozome using *Populus trichocarpa* v3.0 release. A box indicates the *Populus LAC2* gene described in this analysis. Previously characterized *Populus LAC* genes are indicated by name. Newly annotated *LAC* genes in *Populus* are indicated by +.

As a first step to understanding the phylogenetic diversity of *Populus* laccases, we queried the *Populus* and *Arabidopsis* genomes for laccase‐like genes using BLAST in both Phytozome and TAIR databases (Goodstein *et al*., 2012; Huala *et al*., 2001). A total of 17 *Arabidopsis* laccases were found, as previously described (McCaig *et al*., 2005; Turlapati *et al*., 2011), along with 53 *Populus* laccases, 49 of which were previously described utilizing an earlier draft of the *Populus trichocarpa* genome (Lu *et al*., 2013). Utilizing the new draft annotation, we identified four additional laccases distributed across different subfamily categories (Figure 1).

Utilizing the 53 *Populus* and the 17 *Arabidopsis* laccases and a cupredoxin‐like gene as an out‐group, an amino acid‐based phylogeny was constructed for the *Populus* and *Arabidopsis* laccases based on the neighbour‐joining method (Figure 1). The tree created from this analysis places the *Populus* laccases in generalized subfamilies relative to previously reported *Arabidopsis* laccase phylogeny (McCaig *et al*., 2005; Turlapati *et al*., 2011). That is, *Arabidopsis* laccases have been clustered into six arbitrary subfamilies with the expanded *Populus* laccases distributed fairly equally across all subfamilies. *PtLAC3*, which was previously shown to affect xylem fibre cell wall integrity (Ranocha *et al*., 2002), is placed in subfamily II (Figure 1). *AtLAC4* and *AtLAC11* are also found within subfamily II and, when disrupted together with *At*LAC17, completely abolished lignin accumulation in *Arabidopsis* (Zhao *et al*., 2013). *Pt*LAC3 shows highest homology to *Pt*LAC2 (i.e. 90% amino acid identity).

To characterize the protein domain structure of *Pt*LAC2, we identified the closest related laccases from a number of higher plant species including *Manihot esculenta* (Cassava), *Gossypium raimondii* (cotton), *Eucalyptus grandis*,* Medicago truncatula*,* Arabidopsis thaliana*,* Solanum tuberosum* (potato), *Oryza sativa* (rice) and *Zea mays* (corn). Sequences were derived from BLAST search performed from genomes available in Phytozome (http://phytozome.jgi.doe.gov/pz/portal.html), and alignments were based on amino acid sequence (Figure S1). Closer examination of the encoded protein domains of *Pt*LAC2 and its homologs indicates that these homologs all contain the four conserved copper‐binding regions (CBR), including all ten histidines and one cysteine embedded in the CBR I (HWHG) [position 108–111 based on *Pt*LAC2], CBR II (HAH) [position 153–155], CBR III (HP × HLH) [position 506–512] and CBR IV (HCH) [position 568–570] (Figures S1 and S2). Analysis of conservation of the CBR for all *Populus* laccases indicated all contain these conserved motifs except Potri.015G040800 which appears to be missing the N‐terminal region of the protein including CBR I (Figure S2). *PtLAC2* does contain a predicted N‐terminal signal sequence between residues 1 and 23 and a predicted cleavage site between residues 23 and 24, placing this laccase in the secretory pathway (http://www.cbs.dtu.dk) (Petersen *et al*., 2011). Additional analysis of all *Populus* laccases with respect to the presence of signal sequence indicated all laccases except for four (*Pt*LAC3, Potri.005G200600, Potri.005G200500 and Potri.015G040800) contained a predicted signal sequence.

**Figure 2:**
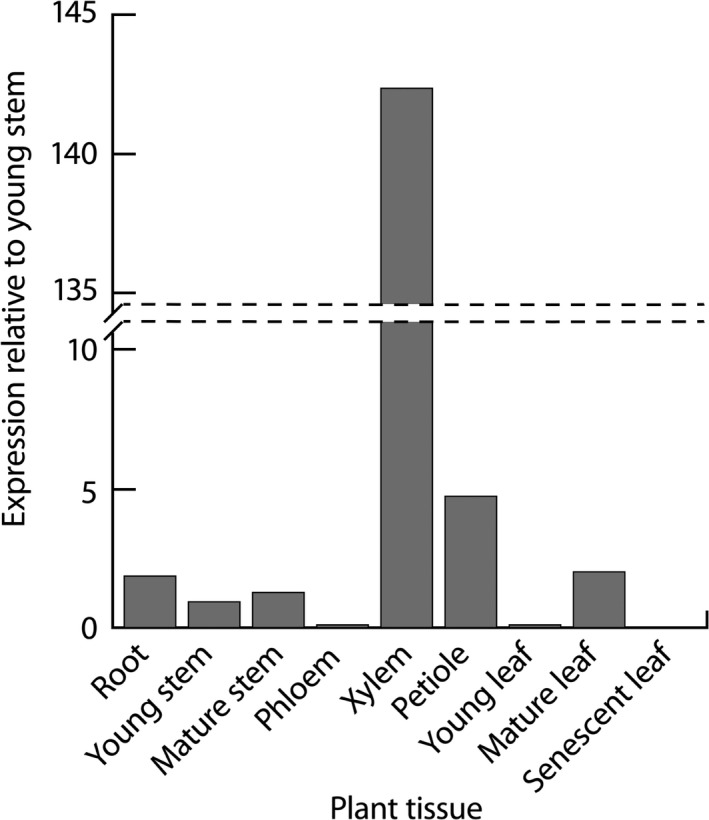
Expression of *PdLAC2* across *Populus deltoides* tissue types. Relative fold expression was calculated using ΔΔ*C*_t_ relative to young stem.

In our qRT‐PCR analysis, *LAC2* had the highest expression in xylem tissue compared to other analysed tissues from *Populus deltoides* (Figure 2). Based on previous expression analysis of *Populus* laccases (Lu *et al*., 2013), all laccase paralogs clustering in subfamily II also showed high xylem expression with the exception of Potri.001G248700 which showed relatively low xylem expression compared to other analysed tissues. The overlapping expression profiles of the *Populus* laccases and sequence similarities suggest there may be functional redundancy within this group.

### Reduction in *PtLAC2* expression leads to altered growth phenotypes in *Populus*

**Figure 3:**
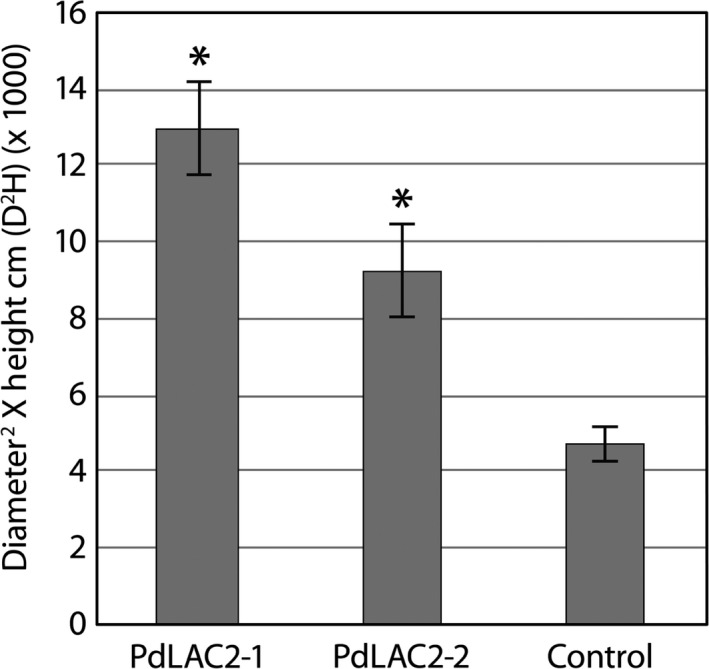
Estimated above‐ground biomass of transgenic *Populus* samples. Above‐ground biomass was estimated using the formula Diameter^2^ × Height cm (D^2^H). *Significant compared to the control, *P*‐value ≤0.01.

**Figure 4:**
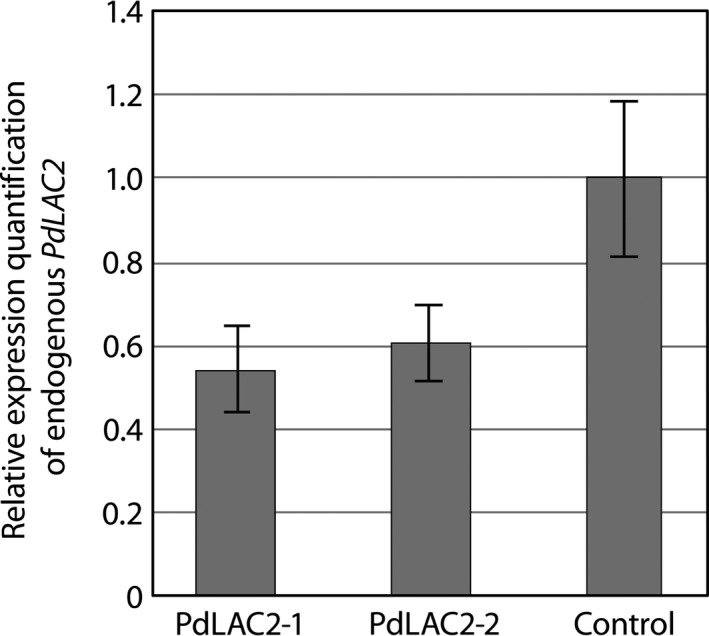
Relative gene expression of endogenous *Pd*LAC2 in RNAi transgenic lines. *PdLAC2‐1* and *PdLAC2‐2* show reduced expression of endogenous *PdLAC2* by 50% and 40%, respectively, compared to control plants.

Previous analysis of *Populus* laccases based on antisense expression of *PtLAC1*,* PtLAC3*,* PtLAC90* and *PtLAC110* did not show any change in lignin quantity or composition which was attributed to functional redundancy or specialized function outside of lignin biosynthesis (Ranocha *et al*., 2002). The only observed defects were observed in *PtLAC3* antisense lines which showed deformed xylem fibre cell walls and an accumulation of undefined phenolics preferentially in xylem (Ranocha *et al*., 2002). To gain further understanding of the function of laccases in subfamily II in *Populus*, we created transgenic lines expressing an RNAi fragment which specifically targeted *LAC2* in *P. deltoides*. This genetic background was utilized for the ease of transformation. The RNAi fragment was designed using the 3′ UTR of *PdLAC2*, and expression was driven by the *UBIQUITIN3* constitutive promoter. Eight independent transgenic lines were generated for analysis. Here, we present results for the two top performing lines, *PdLAC2‐1 and PdLAC2‐2*. Analysis of transgenic lines compared to empty vector control plants showed an increase in above‐ground biomass in the two lines, as measured by diameter^2^ × height (D^2^H) (Figure 3). Utilizing single stem biomass has previously been shown to provide an estimation of above‐ground biomass (Crow, 1978; Ter‐Mikaelian and Korzukhin, 1997; Tuskan and Rensema, 1992). Besides the significant increase in growth, no other developmental or anatomical phenotype was observed in these transgenic lines. To confirm that the biomass phenotype was consistent with a reduction in transcript level due to overexpression of *PdLAC2* RNAi fragment, qRT‐PCR analysis was performed on these lines and the level of *Pd*LAC2 endogenous expression was determined. Three independent empty vector control lines were pooled together and represented in the analysis as control. Both *PdLAC2* RNAi lines showed a reduction in *PdLAC2* transcript by 40% and 50%, for *PdLAC2‐1* and *PdLAC2‐2*, respectively (Figure 4), confirming a reduction in *PdLAC2* transcript in the RNAi transgenic lines.

### Reduction in *LAC2* transcript leads to alteration in S/G ratio although not total lignin quantity

**Figure 5:**
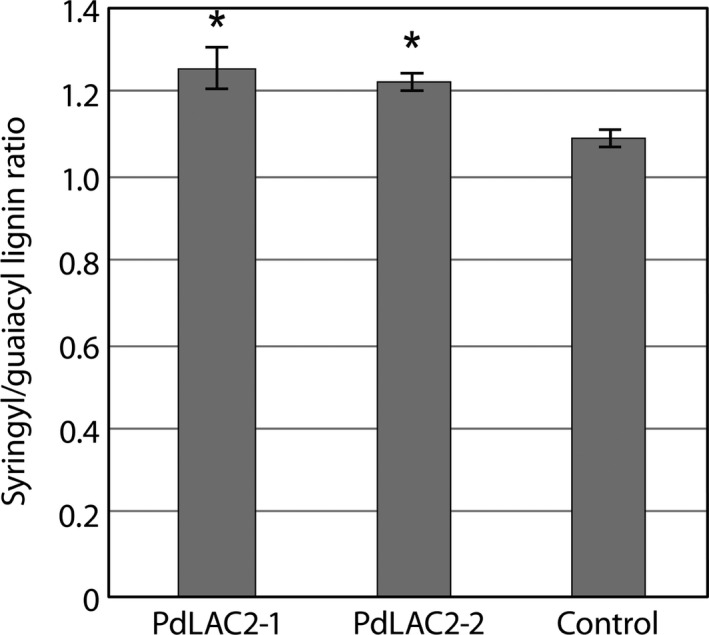
Syringyl/guaiacyl lignin ratio in *PdLAC2* RNAi transgenic lines. Both *PdLAC2‐1* and *PdLAC2‐2* show an increase in S/G ratio compared to control lines. *Significant compared to the control, *P*‐value < 0.01.

Based on molecular beam mass spectrometry (MBMS) measurements from *PdLAC2* RNAi lines, there were no detectable decrease in lignin content for either of the transgenic lines compared to the empty vector controls (Figure S3). However, the two transgenic knock‐down *PdLAC2* lines showed a significant increase in S/G lignin ratio (Figure 5). Specifically, the transgenic lines showed an increase leading to 1.26 and 1.22 S/G ratios, respectively, compared to 1.10 for control lines.

### Reduction in *PdLAC2* transcript leads to changes in metabolite profiling

To explore how reduction in *PdLAC2* may affect cell wall‐related metabolite profiles, we conducted metabolomics analysis using developing xylem tissues from *PdLAC2‐1* and *PdLAC2‐2* lines (Table 1). Both RNAi lines contained reduced monosaccharides concentrations, including glucose, galactose and fructose, yet no significant effect on sucrose. The organic acids, malic acid, fumaric acid and oxalomalic acid were reduced in both lines, and succinic acid was also reduced in *PtLAC2‐1*, although other organic acids, including maleic acid and α‐ketoglutaric acid, were not affected. Citric acid and aconitic acid were increased in *PtLAC2‐1*. Both mono‐ and digalactosylglycerol were elevated in both RNAi lines. *PtLAC2‐1* also had a large number of known and partially identified phenolic glycosides that accumulated, including coumaroyl and caffeoyl glycoside conjugates, several flavonoids, modified carbohydrates (e.g. dehydro, anhydro and methylated sugars) that were conjugated to aromatic metabolites, salicortin and its degradation product 6‐hydroxy‐2‐cyclohexenone‐1‐carboxylic acid. Among the largest accumulations observed were a 4.88‐fold increase in a late‐eluting (19.10 min) coumaric acid rhamnosylglucoside and a 2.81‐fold increase in a dicaffeoyl shikimic acid conjugate that together are indicative of irregular cell wall assembly. Interestingly, coniferin was the only monolignol glucoside that increased, whereas syringin was unchanged, as were the detectable guaiacyl and syringyl lignans.

**Table 1:** Metabolite profile of selected high‐performing *PdLAC2*RNAi lines compared to transgenic control

Metabolites(Name or retention time, key *m*/*z*)	*PdLAC2‐1*		*PdLAC2‐2*	
	Fold change	*P*‐value	Fold change	*P*‐value
19.10 331 171 coumaric acid rhamnosylglucoside	4.88	0.000	1.16	0.633
digalactopyranosylglycerol	2.84	0.000	1.60	0.058
19.88 171 331 463 dicaffeoylshikimic acid conjugate	2.81	0.001	0.95	0.902
citric acid	1.99	0.001	1.20	0.349
14.01 180 165 glycoside	1.96	0.000	0.85	0.320
1,6‐anhydroglucose	1.95	0.000	0.96	0.689
10.37 + 10.70 411 391 modified sugar	1.87	0.000	1.01	0.943
16.12 648 559 leucocyanidin‐like	1.84	0.076	1.32	0.337
salicortin	1.73	0.015	1.16	0.503
10.49 378 103 129 204 modified sugar	1.68	0.039	1.25	0.300
16.30 369 flavonoid glycoside	1.66	0.003	0.87	0.294
galactopyranosylglycerol	1.64	0.007	1.56	0.011
11.24 450 dehydro sugar	1.64	0.000	0.93	0.275
coniferin	1.62	0.003	0.94	0.700
6‐hydroxy‐2‐cyclohexenone‐1‐carboxylic acid	1.62	0.003	0.93	0.602
8.03 203 218 228	1.61	0.001	0.99	0.924
aconitic acid	1.59	0.047	1.39	0.175
benzoic acid	1.59	0.000	1.01	0.878
16.37 382 glycoside	1.49	0.022	1.29	0.115
20.63 171 feruloyl‐caffeoyl conjugate	1.49	0.101	0.81	0.452
catechin	1.48	0.165	1.15	0.605
salicyl‐salicylic acid‐2‐O‐glucoside	1.41	0.150	0.98	0.948
20.59 171 caffeoyl conjugate	1.37	0.112	0.87	0.533
21.45 171 caffeoyl conjugate	1.32	0.133	1.10	0.576
6‐hydroxy‐2‐cyclohexenone alcohol	1.31	0.268	0.86	0.584
16.45 344 327 glycoside	1.31	0.020	0.72	0.023
α‐salicyloylsalicin	1.26	0.367	0.68	0.221
myo‐inositol	1.23	0.391	1.04	0.858
16.40 456 369 dihydromyricetin‐like	1.20	0.645	1.74	0.070
syringin	1.19	0.303	0.82	0.273
ferulic acid	1.17	0.108	0.88	0.112
16.25 guaiacyl lignan glycoside	1.17	0.200	0.85	0.232
dihydromyricetin	1.09	0.798	1.77	0.039
raffinose	1.07	0.785	0.67	0.153
caffeic acid	1.06	0.525	0.84	0.070
16.55 syringyl lignin glycoside	1.05	0.635	0.90	0.326
16.83 369 guaiacyl lignin glycoside	1.05	0.734	0.87	0.213
17.00 547 457 flavonoid glycoside	1.04	0.739	0.75	0.041
aspartic acid	1.04	0.910	0.53	0.116
13.47 342 299 315 phosphorylated metabolite	1.00	0.950	0.83	0.011
glyceric acid	0.99	0.964	1.04	0.794
linoleic acid	0.94	0.613	0.74	0.003
sucrose	0.94	0.203	0.94	0.178
ascorbic acid glucoside	0.93	0.511	0.80	0.030
α‐ketoglutaric acid	0.88	0.448	0.93	0.654
10.74 325 353 427	0.86	0.000	0.95	0.054
catechol	0.86	0.642	0.92	0.763
quercetin	0.85	0.725	1.45	0.242
5‐oxo‐proline	0.85	0.568	0.67	0.179
glutamic acid	0.84	0.622	0.42	0.057
α‐linolenic acid	0.80	0.174	0.66	0.009
erythronic acid	0.78	0.256	1.09	0.545
α‐tocopherol	0.78	0.212	0.76	0.134
11.38 397 425 471	0.64	0.001	0.86	0.101
salicyl alcohol	0.63	0.137	0.79	0.312
11.14 397 425 471	0.61	0.000	0.91	0.197
11.30 397 425 471	0.61	0.000	0.9	0.156
9.94 174 N metabolite	0.58	0.012	0.64	0.017
malic acid	0.52	0.001	0.74	0.019
11.40 363 273 modified sugar	0.49	0.001	0.69	0.009
serine	0.49	0.096	0.35	0.023
ethyl phosphate	0.47	0.022	0.73	0.151
oxalomalic acid	0.43	0.024	0.51	0.030
maleic acid	0.43	0.193	0.55	0.258
glutamine	0.34	0.247	0.12	0.090
13.26 426 325	0.32	0.143	0.06	0.030
threonic acid	0.31	0.083	0.59	0.231
glucose	0.28	0.027	0.43	0.043
galactose	0.27	0.011	0.62	0.099
12.98 320 305	0.25	0.006	0.38	0.009
fructose	0.24	0.012	0.4	0.024
salicyl‐6‐hydroxy‐2‐cyclohexenone	0.24	0.030	0.37	0.045
asparagine	0.07	0.104	0.04	0.064
ornithine	0.00	0.167	0.00	0.121

### Reduction in *PdLAC2* transcript leads to increased five and six carbon sugar release

**Figure 6:**
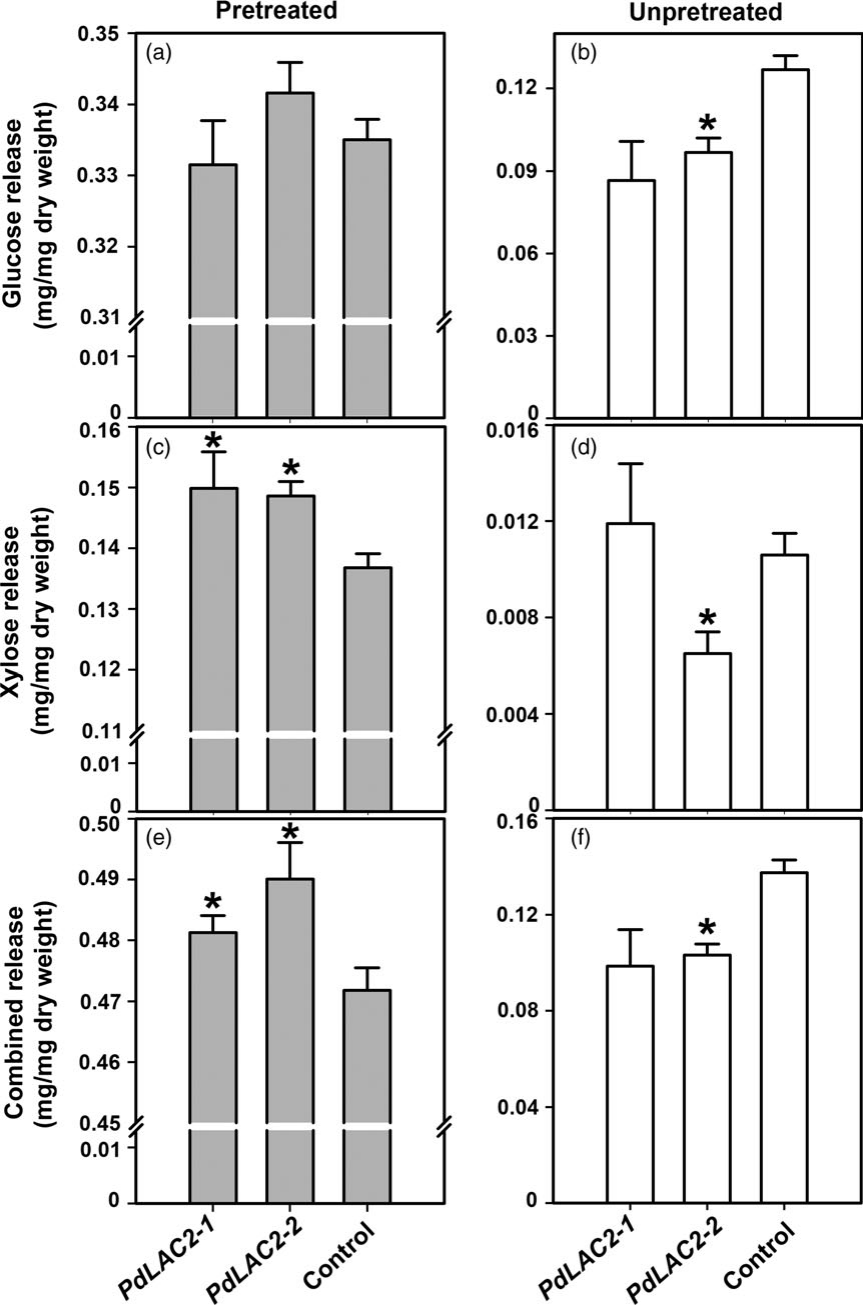
Xylose and glucose release assay of transgenic samples under liquid hot water (LHW) pretreatment and un‐pretreated. Samples were collected with mild LHW pretreatment (a, c, e) and no pretreatment prior to sugar extraction (b, d, f). Rates of xylose release from LHW pretreatment and un‐pretreatment are shown in (a) and (b). Rates of glucose release from LHW pretreatment and un‐pretreatment are shown in (c) and (d). Rates of combined xylose and glucose release are shown in (e) and (f). *Significant compared to the control, *P*‐value < 0.01.

To assess the effect of irregular cell wall assembly on extracting sugars, we evaluated a mild pretreatment condition of hydrothermal, or liquid hot water (LHW), as well as no pretreatment (un‐pretreated) extraction procedure on sugar release. Figure 6 shows the xylose and glucose release from the transgenic samples from both no pretreatment and LHW pretreatment. As expected, biomass treated with LHW showed greater sugar release, a 10‐fold difference, compared to un‐pretreated biomass. This mild pretreatment led to a small but significantly greater release of xylose (Figure 6). However, with no pretreatment, control lines generally showed a greater release of glucose and xylose with the exception of *PdLAC2‐1*. This discrepancy in saccharification of *PdLAC2* RNAi lines compared to controls using the LHW pretreatment vs the un‐pretreated conditions could be attributed to a difference in the manner in which lignin is interacting with the polysaccharides in the cell wall of the *PdLAC2* RNAi lines. That is, there may be structural differences within the cell walls that require some thermal or chemical incubation leading to perturbed recalcitrance with a mild pretreatment.

In order to eliminate the possibility that the *PdLAC2* RNAi lines simply contain more total sugars prior to extraction, we quantified the total carbohydrates for each line including the controls. And in fact, the *PdLAC2* RNAi lines did not contain higher fractions of sugars in cell walls compared to controls (Figure S4). Interestingly, control lines showed higher sugar quantification but with LHW pretreatment still show lower total sugar release thus providing additional evidence for a possible mechanism involving disruption of interlinked structural components in *PdLAC2* knock‐down cell walls.

## Discussion

In this study, we have shown that the reduction in the *PdLAC2* expression through RNAi mediated knock‐down results in biomass with altered cell wall chemistry leading to a pretreatment‐dependent reduction of recalcitrance seen through increased xylose and combined xylose and glucose release. The knock‐down transgenic plants also exhibited increases in S/G ratio and a significant change in metabolite profiles suggestive of an increase in phenolic compounds related to hydroxycinnamoyl glycoside conjugates, salicortin metabolism and flavonoid production. Interestingly, the knock‐down transgenic trees also showed an increase in above‐ground biomass compared to controls. It is not clear why reducing the expression of this laccase would result in increased biomass. One possibility for this phenotype could be attributed to a loosening of the cell wall leading to elongated cells and increased growth as was seen in silencing of GALACTURONOSYLTRANSFERASE 4 (*GAUT4*) in tomato (de Godoy *et al*., 2013) and in *GAUT12* in *Populus* (Biswal *et al*., 2015). In contrast, *Arabidopsis* mutants with loss of function for both laccases *AtLAC4* and *AtLAC17* showed a conditional semi‐dwarf phenotype (Berthet, 2011). Additionally, *AtLAC2* deficient plants show a reduced root elongation developmental defect under stress growth conditions (Cai *et al*., 2006). However, in previous reports, knock‐down of other *Populus* laccases *PtLAC3*,* PtLAC90* and *PtLAC110* had no reported developmental growth defects (Ranocha *et al*., 2002). The precise connection of laccase function and regulation with changes in increased biomass remains unclear although this also reflects the potential divergence of *LAC* gene functionalization and merits further investigation.

The observed reduction in recalcitrance of *PdLAC2* RNAi lines was dependent upon sample pretreatment and not explained by an initial higher quantity of soluble sugars. This discrepancy may point to altered structural components within the *PdLAC2* RNAi plant cell walls without a reduction in lignin content. It is possible these putative extra‐structural components provide novel structures and that maintains cell wall recalcitrance in the absence of thermal or chemical pretreatment. Thus, upon LHW pretreatment, these extra‐structural components foster reduced recalcitrance. Our results suggest that *PdLAC2* is likely involved in higher order interactions of cell wall components. As we only observed an increase in sugar release in knock‐down transgenic lines when samples were subjected to a relatively mild pretreatment condition (Figure 6), we believe the major components of the cell wall have not been disrupted. This can be seen with unchanged total lignin content as well as similar quantities of major sugar components derived through glycosyl composition analysis (Figure S4). However, when energy is added to the *PdLAC2* transgenic samples, through hot water pretreatment, we see a significant increase in sugar release, suggesting that cell wall components are less associated and more amenable to deconstruction. The reduced interactions we postulate occur in the transgenic knock‐down lines would also be consistent with our proposed ‘loosening’ of the cell wall allowing for an increase in biomass in these lines. The increased accumulation of phenolics we observed based on metabolomics analysis may represent the key interfaces of the cell wall components that are regulated by *PdLAC2*. Further investigation into the role of additional laccases and specifically this laccase with respect to biochemical enzymatic activities should yield a continued understanding of elaborate mechanisms regulating cell wall construction. The *PdLAC2* RNAi lines also showed an increase in galactopyranosyl glycerols that may indicate reduced linkages to cell membranes. These accumulations, and those related to salicortin metabolism and hydroxycinnamoyl glycoside conjugate production, suggest a degree of carbon diversion to soluble defence rather than structural (cell wall) components. In non‐pretreated biomass, an increase in phenolic glucosides may prevent extraction of sugars, and under mild LHW pretreatment, phenolics may be solubilized, thus allowing greater access to sugars in the cell wall residue of the transgenic plant biomass.

The presence of expanded laccases in plant genomes advocates for divergence of functions in this gene family. As noted above, *PdLAC2* may be involved in many oxidative reactions, likely involved in these phenolics and possibly downstream reactions linking lignin to other structural cell wall components or simply catalysing the conjugation of flavonoids themselves. The largest metabolite fold change was a late‐eluting coumaroyl rhamnosylglucoside (e.g. rutinose or neohesperidose) conjugate. This accumulation may be an example of the latter, rather than being the result of an unassembled polymer linking component. Such classes of accumulating phenolics, including flavonoids and caffeoylshikimates, are also indicative of altered carbon flux to nonlignin components in the case of the former and upstream storage metabolites associated with the lignin pathway in the case of the latter. Consistent with this hypothesis is the observation that the paralog of *PdLAC2*,* PtLAC3* was shown to be involved in the accumulation of phenolics in xylem tissues (Ranocha *et al*., 2002). It should be noted that reduced expression of *PdLAC2* had a significant impact on S/G lignin ratio that was not seen in knock‐down lines of *PtLAC3*, indicating potential functional divergence between these paralogs. Although speculative, another possibility to explain the selective accumulation of partially identified flavonoids [retention time 16.12 min leucocyanidin‐like (+ *m/z* 648 559) and 16.30 min *m/z* 369 flavonoid glycoside] is that the laccase may play a role in their complexation into the wall, much like the incorporation of tricin into the lignin in cell walls of some monocot species (Lan *et al*., 2015). However, such an analogous flavonoid integration into lignin in a woody dicot species has yet to be demonstrated.

As was shown in the phylogenetic analysis of *Populus* and *Arabidopsis* laccases, there has been a large expansion of *Populus* laccases compared to *Arabidopsis*. *Populus* has substantially larger amounts of secondary cell wall compared to *Arabidopsis*. The more ubiquitous development of secondary cell wall in *Populus* may require a complex set of mechanisms to create a structural and functional cell wall. The expansion and retention of the numerous laccase genes supports the premise of functional or spatial diversification of laccases in *Populus*. A previous analysis of expression of laccase genes in *Populus*, describing a relative spatio‐expression profile of the laccase genes, indicates potential for tissue specific functionalization (Lu *et al*., 2013).

In summation, the above results support a role of *Pd*LAC2 in overcoming plant cell wall recalcitrance beyond correlative reduction in lignin. Modifying alternate components of the plant cell wall, such as changing the associated phenolic composition via manipulation in laccase expression, may provide a novel path to reduced recalcitrance either in conjunction with altered lignin strategies or in lieu of those methods to reduce recalcitrance. There also is the favourable balanced benefit of the use of biomass pretreatment prior to saccharification. While strong acids and other enzymatic pretreatments may allow extraction of more sugars, the negative cost of inputs is a concern. Mild pretreatment strategies, such as LHW, show a greater extraction of sugars even in biomass that may not release as much sugars with no pretreatments. Our results with *PdLAC2* down‐regulation suggest that mild pretreatments increase sugar release and could result in reduction in the cost of lignocellulosic biofuel production.

## Experimental procedures

### Phylogenic and sequence analysis

Protein sequences of 53 *Populus trichocarpa* laccases were collected from Phytozome v10.3 [http://www.phytozome.net/cgi-bin/gbrowse/poplar/]: *Populus trichocarpa* v3.0. Protein sequences of Laccases from *Arabidopsis thaliana* were collected from TAIR [http://www.arabidopsis.org]. All other laccase sequences from other plant species were collected from phytozome. Phylogenetic tree was constructed with neighbour‐joining program using MEGA (Molecular Evolutionary Genetics Analysis) software (Tamura *et al*., 2011). Boostrap values were calculated from 500 independent runs. Sequence alignments were generated using CLC workbench software using neighbour‐joining method (CLC BIO, Aarhus, Denmark). Signal sequences were determined based on TargetP software (www.cbs.dtu.dk/services/TargetP/).

### Generation of transgenic plants

A 201‐bp fragment from the 3'UTR of *PdLAC2* was cloned in the binary vector pAGSM552, deposited in GenBank (KP259613) and used in *Agrobacterium*‐mediated transformation on *Populus deltoides* ‘WV94’ at ArborGen Inc (Ridgeville, SC) as described previously (Biswal *et al*., 2015). A total of eight independent transformation events or lines were obtained, along with five ramets for each transgenic event, together with equal numbers of ramets for empty vector transformed control plants, were propagated at Oak Ridge National Laboratory greenhouses at constant 25 °C and 16‐h day length. All plants were initially grown in Leach tubes and transferred to larger pots, and after six months of growth, plant height and stem diameter were measured, stem samples were collected and air‐dried for cell wall chemistry analyses. Primers used for generating RNAi fragment were as follows: PdLAC2 RNAi F: 5′GTATCGTATAGTCTGAAGATCTGG, PdLAC2 RNAi R: 5′ GGAATCAAAGTGCCAAATCC.

### qRT‐PCR assays

Xylem samples were collected for three ramets each of the two independent transgenic lines and three independent empty vector control plants. RNA was extracted using the Spectrum Plant Total RNA Kit (Sigma, St. Louis, MO) with a slight modification. Such that 850 μL of prewarmed (65 °C) cetyltrimethyl ammonium bromide (CTAB) buffer containing 10 μL of β‐mercaptoethanol (Sigma) was added to 100 mg fresh weight sample, vortexed for 5 min and incubated at 65 °C for 5 min. Then, 600 μL of chloroform : isoamyl alcohol (24 : 1 v:v) was added and supernatant was passed through a filter column (Sigma). The filtrate was diluted with 750 μL of 95% EtOH and passed through Sigma binding column. Sigma protocol was followed including on‐column DNase digestion per manufactures instructions (Sigma). cDNA was created using 1 μg of RNA using Thermo Fisher Scientific 1st strand cDNA synthesis kit according to manufacturer's instructions. The 1st strand reaction was diluted to 200 and 1.4 μL used per reaction for qRT‐PCR analysis. qRT‐PCR was performed using StepOnePlusTM Real‐Time PCR system (Applied Biosystems, Foster City, CA) using SYBR green reaction mix (Bio‐Rad Life Sciences, Hercules, CA) according to manufacturer's recommendations for 20 μL reactions. Gene expression was calculated using ΔΔcT method (Livak and Schmittgen, 2001) using 18s ribosomal subunit for template normalization. Primers used were as follows: 18sqF 5′ AATTGTTGGTCTTCAACGAGGAA, 18sqR 5′ AAAGGGCAGGGACGTAGTCAA, LAC2qF 5′ CTTGCGCTATAAGGGAACCA and LAC2qR 5′CCCGACACCGATAGTGAAGT.

### Molecular beam mass spectrometry assay

Four mg of dried, ground [20/80 mesh] stem biomass was placed into a pyrolysis molecular beam mass spectrometry chamber, and then, using 17 eV electron impact ionization, mass spectral data were acquired on a Merlin Automation data system version 3.0 from 30 to 450 *m/z* (Sykes *et al*., 2009). Lignin estimates were determined as described previously (Sykes *et al*., 2009). S/G ratios were determined by summing the area under the peaks attributed to syringyl moieties (i.e. *m/z* 154, 167, 168, 182, 194, 208 and 210) and dividing this area by the area under the peaks attributed to guaiacyl moieties (i.e. *m/z* 124, 137, 138, 150, 164 and 178).

### Saccharification assay

Biomass was extracted with α‐amylase (Spirizyme Ultra—0.25%) and α‐glucosidase (Liquozyme SC DS—1.5%) in 0.1 m sodium acetate (24 h, 55 °C, pH 5.0) to remove possible starch content (16 mL enzyme solution per 1 g biomass). This was followed by an ethanol (95% v/v) Soxhlet extraction for an additional 24 h to remove extractives. After drying overnight, 5 mg (±0.5 mg) of biomass was weighed in triplicate into one of 96 wells in a solid Hastelloy microtitre plates and 250 μL of water was added. Samples are then sealed with silicone adhesive, Teflon tape. For pretreatment, the samples were reacted at 180 °C for 17.5 min. Once cooled 40 μL of buffer‐enzyme stock was added. The buffer‐enzyme stock was 8% CTec2 (Novozymes, Bagsværd, Denmark) (excess enzyme loading of 70 mg/g biomass) in 1 m sodium citrate buffer. The samples were then gently mixed and left to statically incubate at 50 °C for 70 h. After 70‐h incubation, an aliquot of the saccharified hydrolysate was diluted and tested using megazymes GOPOD (glucose oxidase/peroxidase) and XDH assays (xylose dehydrogenase). Results were calculated using standard curves created from mixtures of glucose and xylose.

### Glycosyl composition and metabolite profiling

Cell wall glycosyl composition analysis was performed by combined gas chromatography/mass spectrometry (GC/MS) of the per‐*O*‐trimethylsilyl (TMS) derivatives of the monosaccharide methyl glycosides produced from the sample by acidic methanolysis as described previously (Santander *et al*., 2013). Briefly, the samples (between 200 and 500 μg) were heated with methanolic HCl in a sealed screw‐top glass test tube for 18 h at 80 °C. After cooling and removal of the solvent under a stream of nitrogen, the samples were treated with a mixture of methanol, pyridine and acetic anhydride for 30 min. The solvents were evaporated, and the samples were derivatized with Tri‐Sil^®^ (Pierce, Waltham, MA) at 80 °C for 30 min. GC/MS analysis of the TMS methyl glycosides was performed on an Agilent 7890A GC interfaced to a 5975C MSD, using an Supelco Equity‐1 fused silica capillary column (30 m × 0.25 mm ID).

For metabolite profiling, 25 mg of actively dividing xylem tissues lyophilized and ground with a Wiley mill were twice extracted from each transgenic line and controls with 2.5 mL 80% ethanol overnight and then the extracts combined prior to drying a 0.50‐mL aliquot in a nitrogen stream. As an internal standard, 75 μL of sorbitol at 1.0 mg/mL was added to the first extract. Dried extracts were dissolved in acetonitrile, followed by TMS derivatization and analysed by GC‐MS, as described elsewhere (Jung *et al*., 2009; Li *et al*., 2012). Metabolite peaks were extracted using characteristic mass‐to‐charge (*m/z*) ratio and quantified by area integration, and the concentrations were normalized to the quantity of the internal standard (sorbitol) recovered and the amount of sample extracted, derivatized and injected. A large user‐defined database of mass spectral electron impact ionization fragmentation patterns of TMS‐derivatized compounds (~2300 signatures) was used to identify the metabolites of interest. Unidentified metabolites were represented by their retention time and key *m/z* ratios. The metabolite data were presented as fold changes of the transgenic line vs. the average of the control lines. Student's *t*‐tests were used to determine whether differences were statistically significant (*P *≤ 0.05).

## Supporting information


**Figure S1** Amino acid alignment of *Populus trichocarpa* laccases. Alignment was created with CLC Main Workbench software (www.clcbio.com). Most common amino acid per position is noted with Sequence logo at the base of alignment. Divergent residues are shaded in grey.
**Figure S2** Amino acid alignment of homologous laccases to *Pt*LAC2 (Potri.008G064000) across different plant species. Homologs were determined based on BLAST search through Phytozome v 10.3 (www.phytozome.jgi.doe.gov). Alignment was created using CLC Main Workbench software. Copper Binding Regions (CBR) are indicated with solid line below residues. Diverged residues are shaded grey.
**Figure S3** Lignin content in *PdLAC2* RNAi transgenic lines. Lignin content was measured through Molecular Beam Mass Spectrometry.
**Figure S4** Carbohydrate analysis of *Pd*LACs transgenic lines and transgenic controls. Quantification is represented as percentage of each carbohydrate per sample.
