# Supplementary material for: Knockdown of a laccase in Populus deltoides confers altered cell wall chemistry and increased sugar release
Source: Plant Biotechnol J. 2016 Apr 15;14(10):2010–20. doi: 10.1111/pbi.12560 (PMC5043505; doi:10.1111/pbi.12560)
Supplement: Supplementary file 1 — Figure S1 Amino acid alignment of Populus trichocarpa laccases. Alignment was created with CLC Main Workbench software (www.clcbio.com). Most common amino acid per position is noted with Sequence logo at the base of alignment. Divergent residues are shaded in grey. Figure S2 Amino acid alignment of homologous laccases to PtLAC2 (Potri.008G064000) across different plant species. Homologs were determined based on BLAST search through Phytozome v 10.3 (www.phytozome.jgi.doe.gov). Alignment was created using CLC Main Workbench software. Copper Binding Regions (CBR) are indicated with solid line below residues. Diverged residues are shaded grey. Figure S3 Lignin content in PdLAC2 RNAi transgenic lines. Lignin content was measured through Molecular Beam Mass Spectrometry. Figure S4 Carbohydrate analysis of PdLACs transgenic lines and transgenic controls. Quantification is represented as percentage of each carbohydrate per sample. [file PBI-14-2010-s001.pdf]

Supporting information

Knockdown of a laccase in *Populus deltoides* confers altered cell wall chemistry and increased sugar release

Anthony C. Bryan[1], Sara Jawdy[1], Lee Gunter[1], Erica Gjersing[2], Robert Sykes[2], Maud A.W. Hinchee[3], Kimberly A. Winkeler[3], Cassandra M. Collins[3], Nancy Engle[1], Timothy J. Tschaplinski[1], Xiaohan Yang[1], Gerald A. Tuskan[1], Wellington Muchero[1,*] and Jin-Gui Chen[1,*]

Supplementary Figure S1

```
                                       20                                      40                                      60
                                        |                                       |                                       |
Potri.008G064000  MENYRAR---  -----AILLL  VIFI--FPAL  VECE----VR  LYDFRVVLTN  TTKLCSTKSI  46
Potri.010G193100  MEYYQAR---  -----T-MLL  VIFI--FPAL  VECK----VR  LYNFRVVLTN  TTKLCSTKSI  45
Potri.001G248700  MV------DM  ALWL-RVLVL  VACL--FPAS  VESM----VR  HYKFNVVMKN  TTRLCSEKPI  47
Potri.009G042500  M-------DM  APWI-RVLVL  VACL--FPAS  VESM----VR  HYKFNVVMKN  STKLCSTKPI  46
Potri.016G112100  MIRKSAKMEY  SWF--RFMLL  AVSL--FPAL  VECR----VR  HYKFNVVMKN  TTRLCSSKPV  52
Potri.016G112000  M-------EY  SCF--RFMLL  AVCL--FPAV  VECR----IR  HYKFNVVMKN  TTRLCSSKPI  45
Potri.006G097000  M-------EY  SNLLIRFMLL  AVCL--LPAL  VECR----IR  HYKFNVVMKN  TTRLCSRKPI  47
Potri.006G096900  M-------EY  ACWL-RFMLL  AVCL--FPAL  VQCR----VR  HYKFNVVMKN  TTRLCSRKPI  46
Potri.006G097100  M-------EY  SNWLIRFMLL  AVCL--LPAL  VECR----IR  HYKFNVVMKN  TTRLCSRKPI  47
Potri.007G023300  MVS--SRGFL  SWLIFLFIGI  LGFIP-FPAE  AA------IK  KYQFDIQVKN  VSRLCHAKPI  51
Potri.009G102700  MAAALSKKLC  WASYILYLY-  --FIY-HPAE  AA------VK  RYQFDIQVKN  VSRLCHAKPI  50
Potri.004G156400  MAAALSKSFC  LGYYFLLLCL  IGFIS-HPAK  AA------VK  KYLFDIQVKN  VSRLCHAKPI  53
Potri.006G087500  MGSAFLRILV  TALCALWIFS  ELVVAKHA--  --G----ITR  HYKFDIKLQN  VTRLCRTKSI  52
Potri.009G156800  MGAS-VPASP  EILLTILLFA  MSCLWAFPEV  AGAKHAGITR  HYKFNIKLTN  VTRLCHTKSM  59
Potri.009G156600  MGAS-VPASP  EILLTILLFA  MSCLWAFPEV  AGAKHAGITR  HYKFNIELTN  VTRLCHTKSM  59
Potri.001G184300  MGAS-VPASP  EILLTILLFA  MSCLWAFPEV  AGAKHAGITR  HYKFNIKLKN  VTRLCHTKSM  59
Potri.009G034500  MGAP-VPASP  GILLTILLFA  MSCLWAFPEV  AGAKHAGITR  HYKFNIKLTN  VTRLCHTKSM  59
Potri.006G087100  MGNSPRSTVL  PSMAALQLLC  FFFFSLVPDF  AAA----ITR  QYTFNITHKN  FTRLCHTRSL  56
Potri.001G341600  MGASILP--P  PAFRAL-LFS  FSIFCLLPEH  AFA----VTR  HYKFDIKLQN  VTRLCHSKSM  53
Potri.001G054600  MGVSFLP--S  PAFLGLLLFS  FVTLSLHPKP  AVA----TTR  HYKLDVMLQN  VTRLCHTKSM  54
Potri.001G401300  MGASFLP--S  PAFLAVFLIS  FVTLSIHPEP  ALA----ITR  HYKFDVMLQN  VTRLCHTKSI  54
Potri.001G401100  MGVYLLP--S  PASLAVFLSS  FVTLFVHPRP  AIA----ITR  HYKFDVLLQN  VTRLCHTKSM  54
Potri.011G120300  MGASLLP--P  PAFLAVFLFS  FVTLSVNPEP  ALA----ITR  HYKFDVMLQN  VTRLCHTRSM  54
Potri.011G120200  MGASLLP--P  PAFLAVFLFS  FVTLSVNPEP  ALA----ITR  HYKFDVMLQN  VTRLCHTKSM  54
Potri.015G040700  M---------  EGVRKHYGIL  LASLAIIAAA  LPCCSSQTTR  GFQFNVEWKK  VTRLLTPSN-  50
Potri.015G040800  M---------  S---KNF---  ----------  ----------  ----------  ----------  5
Potri.015G040600  M---------  EAVRKHYGIL  LASLAIIAAA  LPCCSSETTR  RFQFNVEWKK  VTRLCTTKQL  51
Potri.015G040400  M---------  EGVRKHYGIL  LASLAIIAAA  LPCCSSQTTR  RFQFNVEWKQ  VTRLCTTKQL  51
Potri.012G048900  M---------  EGGHKNSGIL  LVSLVIIAGA  LPFCSSQATR  RFQFNVEWKK  VTRLCTTKQL  51
Potri.016G107900  M---------  ---LRLLFLL  TCALALLASS  VASAAIVE--  -RSFYVKNLT  VRRLCSEQVV  45
Potri.016G106100  M---------  ---LRLLFLL  TCALALLASS  VASAAIVE--  -RSFYVKNLT  LRRLCSEQVV  45
Potri.016G106300  M---------  ---LRLLFLL  TCALALLASS  VASAAIVE--  -HSFYVKNLT  VRRLCSEQVV  45
Potri.016G107500  M---------  ---SRLLFLL  TCALALLASS  VASAAIVE--  -HSFYVQNLT  VRRLCSEQVV  45
Potri.016G106000  M---------  ---SRLLFLL  TCALALLASS  VASAAIVE--  -HSFYVQNLT  VRRLCSEQVV  45
Potri.006G094100  M---------  ---LRLLFWL  TCALVLLASS  VASAAIVE--  -HSFYVKNLT  VRRLCTEQVV  45
Potri.019G121700  METH---N--  LTVKQLYCCL  LLSIFVIISF  QASSSEPETH  YHEFVIQAKP  VRRLCRTHNT  55
Potri.019G124300  METH---N--  LTVKQLYCCL  LLSIFVIISF  QASSSEPETH  YHEFVIQAKP  VRRLCRTHNT  55
Potri.013G152700  METR---N--  LTVKQVSYCL  FLSIFVIFSF  QAHFSEAETH  YREFVIQAKP  VKRLCRTHNT  55
Potri.010G183600  MEVI---KSI  FADRH--CSF  FL-VVLLLAS  TMSLAIAEIH  HHDFVVQATK  VKRLCKTHNS  54
Potri.008G073700  MEVI---NRI  FANRH--CSF  FL-L-LLLAS  AMSLAIAKTH  HHDFTVQATK  VKRLCKTHNS  53
Potri.008G073800  MEGF---DNI  FAINHRLSLF  FLGLLLLLAS  ALSLANAETH  NHDFVIQATP  VKRLCKTQNS  57
Potri.010G183500  MEVF---NNI  FAINHRCSSF  FLGLLLLLAS  ALSLANAKSH  YHDFVIQATP  VKRLCKTQNS  57
Potri.014G100600  MA-----NLV  TSFMLWLCLI  SYAYTTIHAA  PEWPRGRSTR  FYDFKIQTMT  VNKLCNSKQI  55
Potri.019G088900  MGIS------  ---RLGFIAG  LIWFMAMDWQ  GLCMAQSNVH  RYNFVLQNAQ  FTRLCETKTM  51
Potri.019G088800  MGIS------  ---RLGFIAG  LIWFMAMDWQ  GLCMAQSSVH  RYNFVLQNAQ  FTRLCETKTM  51
Potri.019G088600  MGIS------  ---RLGFIAG  LIWFMAMDWQ  GLCMAQSNVH  RYNFVLQNAQ  FTRLCETKTM  51
Potri.019G088700  MGIS------  ---RLGFIAG  LIWFMAMDWQ  GLCMAQSNVH  RYNFVLQNAQ  FTRLCETKTM  51
Potri.019G088500  MGIS------  ---RLGFIAG  LIWFMAMDWQ  GLCMAQSSVH  RYNFVLQNAQ  FTRLCETKTM  51
Potri.001G206200  MVKI------  ---LRLLGFI  VS--LII--Q  NYTTANGKIH  HHKFVVKSAS  FTRLCNTKEI  47
Potri.005G200700  MLFC------  ---KKTLVFK  ILWVLLF--F  S-VHCLAATH  YH-FKVMEAP  YTRLCSKKKI  47
Potri.011G071100  M---------  ----RVLAFQ  ISRFLLF--G  GFLCCQAIVH  HT-FVVKDVP  YTRLCSTKNI  44
Potri.005G200600  MMAI------  ---NRVLAFQ  ILRFLLF--G  GFLCCQAIVH  HT-FVVKDVP  YTRLCSTKNI  48
Potri.005G200500  MIPI------  ---MRVLAFQ  ILRFLLF--G  GFLCCQAIVH  HT-FVVKDVP  YTRLCSTKNI  48
```

Sequence logo (4.3bits / 0.0bits)

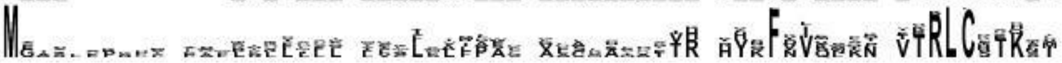

Supplementary Figure S1 (cont.)

	80		100		120	

Potri.008G064000	VTINGKFPGP	TIYAREGDNV	NIKLTNHVQY	NVTIHWHGVR	QLRTGWSDGP	AYITQC-PIR	105
Potri.010G193100	PTINGKFPGP	TIYAREGDNV	NIRLTNQVQY	NVTVHWHGVR	QLRTGWADGP	AYITQC-PIQ	104
Potri.001G248700	VTVNGRFPGP	TLVAREDDTV	LVKVVNHVKY	NVSIHWHGIR	QLRTGWADGP	AYITQC-PLQ	106
Potri.009G042500	VTVNGQFPGP	TLVAREDDTV	LVKVVNHVKY	NVSIHWHGIR	QLRTGWADGP	AYITQC-PIQ	105
Potri.016G112100	VTVNGRFPGP	TLYAREDDTV	LVKVVNHVKY	NVSIHWHGIR	QLRTGWADGP	AYITQC-PIQ	111
Potri.016G112000	VTVNGLFPGP	TLYAREDDTV	LVKVVNRVKY	NLSIHWHGIR	QLRTGWADGP	AYITQC-PIQ	104
Potri.006G097000	VTVNGRFPGP	TLYAREHDTV	LVKVVNHVKY	NVSIHWHGIR	QLRTGWADGP	AYITQC-PIQ	106
Potri.006G096900	VTVNGRFPGP	TLYAREDDTV	LVKVVNHVKY	NVSIHWHGIR	QLRTGWADGP	AYITQC-PIQ	105
Potri.006G097100	VTVNGRFPGP	TLYAREHDTV	LVKVVNHVKY	NVSIHWHGIR	QLRTGWADGP	AYITQC-PIQ	106
Potri.007G023300	VTVNGRFPGP	TIYVREGDRV	MVNVTNYAQY	NMSIHWHGLK	QYRNGWADGP	AYITQC-PIQ	110
Potri.009G102700	VTVNGRFPGP	TVYVREGDRV	LVNVTNHARY	NMSIHWHGLK	QFRNGWADGP	AYITQC-PIK	109
Potri.004G156400	VTVNGRFPGP	TVYVREGDRV	QVNVTNHAKY	NMSIHWHGLK	QFRNGWADGP	AYITQC-PIK	112
Potri.006G087500	VTVNGQIPGP	RIIAREGDRL	LIKVVNHVQY	NVTLHWHGIR	QLRSGWADGP	AYVTQC-PIQ	111
Potri.009G156800	VTVNGKFPGP	RVVAREGDRL	VVKVVNHVPN	NISIHWHGIR	QLQSGWADGP	AYITQC-PIQ	118
Potri.009G156600	VTVNGKFPGP	RVVAREGDRL	VVKVVNHVPN	NISIHWHGIR	QLQSGWADGP	AYITQC-PIQ	118
Potri.001G184300	VTVNGKFPGP	RVVAREGDRL	VVKVVNHVPN	NISIHWHGIR	QLQSGWADGP	AYITQC-PIQ	118
Potri.009G034500	VTVNGKFPGP	RVVAREGDRL	VVKVVNHVPN	NISIHWHGIR	QLQSGWADGP	AYITQC-PIQ	118
Potri.006G087100	VTVNGQFPGP	RLVAREGDQV	LVKVVNHVAE	NITIHWHGVR	QLTSGWADGP	AYVTQC-PIQ	115
Potri.001G341600	VTVNGQFPGP	RIVAREGDNL	FIKVVNHVQN	NISIHWHGIR	QLQSGWADGP	AYITQC-PIQ	112
Potri.001G054600	VTVNGKFPGP	RIVAREGDRL	LIKVVNHVQN	NISIHWHGIR	QLRSGWADGP	AYVTQC-PIQ	113
Potri.001G401300	VTVNGKFPGP	RIVAREGDRL	LIKVVNHVQN	NISIHWHGIR	QLRSGWADGP	AYITQC-PIQ	113
Potri.001G401100	VTVNAKFPGP	CIVAREGDRL	LIKVVNHVQN	NISIHWHGIR	QLRSGWADGP	AYVTQC-PIQ	113
Potri.011G120300	VTVNGKFPGP	RIVAREGDRL	VIRMVNHVQN	NISIHWHGIR	QLRSGWADGP	AYVTQC-PIQ	113
Potri.011G120200	VTVNGKFPGP	RIVAREGDRL	VITVVNHVQN	NISIHWHGIR	QLRSGWADGP	AYVTQC-PIQ	113
Potri.015G040700	----------	------GDSI	HIKVKNRIAQ	NTTLHWHGVR	QLRTGWADGP	AYVTQC-PIR	93
Potri.015G040800	----------	----------	----------	----------	----------	----QCLKIR	11
Potri.015G040600	LMVNGQYPGP	TIAVHEGDNV	EIKVKNRIAQ	NTTLHWHGVR	QLRTGWADGP	AYITQC-PIR	110
Potri.015G040400	LMVNGQYPGP	TIAVHEGDNV	EINVKNQIAQ	NTTLHWHGVR	QLRTGWADGP	AYVTQC-PIR	110
Potri.012G048900	LTVNGQYPGP	TIAVHEGDRV	EIKVKNRIAH	NTTLHWHGLR	QLRTGWADGP	AYITQC-PIR	110
Potri.016G107900	TAVNGSLPGP	TLRVREGDTL	IVHVFNKSPY	DLSIHWHGVF	QLLSAWADGP	SMVTQC-PIT	104
Potri.016G106100	TAVNGSLPGP	TLRVREGDTL	IVHVFNKSPY	DLSIHWHGVF	QLLSAWADGP	SMVTQC-PIT	104
Potri.016G106300	TAVNGSLPGP	TLRVREGDTL	IVHVFNKSPY	NLTIHWHGVF	QLLSAWADGP	SMVTQC-PIT	104
Potri.016G107500	TAVNGSLPGP	TLRVREGDTL	IVHVFNKSPY	NLTIHWHGVF	QLLSAWADGP	SMVTQC-PIP	104
Potri.016G106000	TAVNGSLPGP	TLRVREGDTL	IVHVFNKSPY	NLTIHWHGVF	QLLSAWADGP	SMVTQC-PIP	104
Potri.006G094100	TAVNGSLPGP	TLRVQEGDTL	KVHVFNKSPY	NMTLHWHGVF	QLLSAWADGP	NMVTQC-PIP	104
Potri.019G121700	ITVNGLFPGP	TLEVRDGDTL	VIKAINNARY	NVTLHWHGVR	QLRNPWADGP	DRVTQC-PIQ	114
Potri.019G124300	ITVNGLFPGP	TLEVRDGDTL	VIKAINNARY	NVTLHWHGVR	QLRNPWADGP	DRVTQC-PIQ	114
Potri.013G152700	ITVNGLFPGP	TLEVRDGDTL	VIKAVNNARY	NVTLHWHGIR	QLRNPWADGP	DRVTQC-PIR	114
Potri.010G183600	ITVNGMFPGP	TLEVKNGDTL	VVKVVNKARY	NVTIHWHGIR	QMRTGWADGP	EFVTQC-PIR	113
Potri.008G073700	ITVNGMFPGP	TLEVKNGDTL	VVKVVNRARY	NVTIHWHGIR	QMRTGWADGP	EFVTQC-PIR	112
Potri.008G073800	ITVNGMFPGP	TLEVNNGDTL	VVNVVNKAQY	NVTIHWHGVR	QMRTGWADGP	EFVTQC-PIR	116
Potri.010G183500	ITVNGMFPGP	TLEVNNGDTL	VVNVVNKARY	NVTIHWHGIR	QMRTGWADGP	EFVTQC-PIR	116
Potri.014G100600	VTVNNMFPGP	VVYAQQGDRL	IVKVSNESPY	NATIHWHGVR	QILSCWFDGP	SYITQC-PIQ	114
Potri.019G088900	LTVNGSFPGP	TIHARRGDTI	YVNVHNEGDY	GVTIHWHGVK	QPRNPWSDGP	ENITQC-PIQ	110
Potri.019G088800	LTVNGSFPGP	TIHARRGDTI	YVNVHNEGDY	GVTIHWHGVK	QPRNPWSDGP	ENITQC-PIQ	110
Potri.019G088600	LTVNGSFPGP	TIHARRGDTI	YVNVHNEGDY	GVTIHWHGVK	QPRNPWSDGP	ENITQC-PIQ	110
Potri.019G088700	LTVNGSFPGP	TIHARRGDTI	YVNVHNEGDY	GVTIHWHGVK	QPRNPWSDGP	ENITQC-PIQ	110
Potri.019G088500	LTVNGSFPGP	TIHARRGDTI	YVNVHNEGDY	GVTIHWHGVK	QPRNPWSDGP	ENITQC-PIQ	110
Potri.001G206200	LTVNGKFPGP	TLEAYTGDEL	IVTVYNRAKY	NITLHWHGAR	QVRNPWSDGP	EYITQC-PIQ	106
Potri.005G200700	LTVNGQFPGP	ALHVHHGDTI	YVTVHNKGRY	NITIHWHGVK	LTGYPWSDGP	EYITQC-PIQ	106
Potri.011G071100	MTVNGQFPGP	TLYVTKGETI	IVDVINKSPH	NITIHWHGVK	QPKYPWSDGP	EYITQC-PIQ	103
Potri.005G200600	MTVNGQFPGP	TLYVTKGETI	IVDVINKSPH	NITIHWHGVK	QPKYPWSDGP	EYITQC-PIQ	107
Potri.005G200500	MTVNGQFPGP	TLYVTKGETI	IVDVINKSPH	NITIHWHGVN	QPKYPWSDGP	EYITQC-PIQ	107

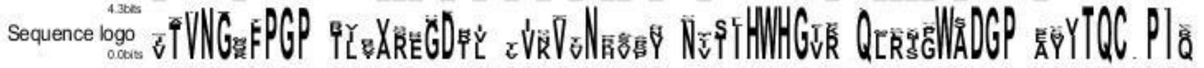

Supplementary Figure S1 (cont.)

```
                          140                                 160                                 180
                           |                                   |                                   |
Potri.008G064000 PGQSYLYNFT LTGQRGTLLW HAHISWLRAT IHGAIVILPQ -KGVPYPFPK P-DKEKIIIL 163
Potri.010G193100 PGQSYLYNFT LTGQRGTLLW HAHISWLRAT IHGAIVIFPK -KGVPYPFPK P-DKEKIIIL 162
Potri.001G248700 PGQNFVYNFT ITGQRGTLLW HAHILWLRAT VHGGIVILPK -RGVPYPFPT P-HREEVIVL 164
Potri.009G042500 PGQSFVYNFT ITGQRGTLLW HAHILWLRAT VHGAIVILPK -RGVPYPFPT P-RKEKVIIL 163
Potri.016G112100 TGQSYVYNFT ITGQRGTLLW HAHILWLRAT VHGAIVVLPK -RGVPYPFPA P-HKEFVVVL 169
Potri.016G112000 PGQSYVYNFT ITGQRGTLLW HAHILWLRAT VHGALVVLPK -LGVPYPFPA P-HKEVVVVL 162
Potri.006G097000 PGQSYVYNFT ITGQRGTLLW HAHILWLRAT VHGALVVLPK -RGIPYPFPA P-HKEVLVVL 164
Potri.006G096900 PGQSYVYNFT ITGQRGTLLW HAHILWLRAT VHGAMVVLPK -RGIPYPFPG P-HKEVVVVL 163
Potri.006G097100 PGQSYVYNFT ITGQRGTLLW HAHILWLRAT VHGAMVVLPK -RGIPYPFPA P-HKEVVVVL 164
Potri.007G023300 TGSSYTYDFN VTGQRGTLWW HAHILWLRAT VYGAIVIMPK -QGTPYPFPQ P-NMEVPILL 168
Potri.009G102700 TGHSYTYDFN VTGQRGTLWW HAHILWLRAT VYGAIVIMPK -PGTPFPFPQ P-HREEIIIF 167
Potri.004G156400 TGHSYTYDFN VTGQRGTLWW HAHIFWLRAT VYGAIVIMPK -PGTPFPFPQ P-HREETIIL 170
Potri.006G087500 TGQSYVYNFT VTGQRGTLFW HAHISWLRAT LYGPIVILPK -KGVSYPFPL P-HKEVPIIF 169
Potri.009G156800 TNQTYVYNFT ITGQRGTLFW HAHLSWLRAS VYGPLIILPK -RNVSYPFAK P-HKEVTIML 176
Potri.009G156600 TNQTYVYNFT ITGQRGTLFW HAHLSWLRAS VYGPLIILPK -RNVSYPFAK P-HKEVTIML 176
Potri.001G184300 TNQTYVYNFT VTGQRGTLFW HAHLSWLRAS VYGPLIILPK -RNVSYPFAK P-HKEVTIML 176
Potri.009G034500 TNQTYVYNFT VTGQRGTLFW HAHLSWLRAS VYGPLIIFPK -RNVSYPFAK P-HKEVTIML 176
Potri.006G087100 TGQAYTYNFT ITGQRGTLLW HAHISWLRSS LYGPIIILPK -LNESYPFKK P-YKEIPILF 173
Potri.001G341600 TGQSYVYNYT IVGQRGTLWW HAHISWLRST VYGPLIILPK -RGVQYPFAK P-YKEVPIIF 170
Potri.001G054600 TGQSYVYNYT IVGQRGTLWW HAHISWLRST LYGPLIILPK -LGTPYPFVK P-YKEVPIIF 171
Potri.001G401300 TGQSYVYNYT IVGQRGTLWW HAHISWLRST LYGPLIILPK -LGTTYPFAK P-HKEVPIIF 171
Potri.001G401100 TGQSYVYNYT IVGQRGTLWW HAHISWLRST LYGPLIILPK -LGTPYPFAK P-DKEVPIIF 171
Potri.011G120300 TGQSYVYNYT IVGQRGTLWW HAHISWLRST LHGPIILLPK -LGTPYPFAK P-YKEVPIIF 171
Potri.011G120200 TGQSYVYNYT IVGQRGTLWW HAHISWLRST LYGPIILLPK -LGTPYPFAK P-YKEVPIIF 171
Potri.015G040700 GGQSYTYKFT VTGQRGTLLW HAHYAWQRAS VYGAFIIYPR ---IQYPFSH RIQAEIPIIF 150
Potri.015G040800 GGHSYTYKFT VTGQRGTLLW HAHYAWQRAS VYGAFIIYPR ---IPYPFSH PIQAEIPIIF 68
Potri.015G040600 GGQSYTYKFT VTGQRGTLLW HAHYAWQRAS VYGALIIYPR ---IPYPFSH PIQAEIPIIF 167
Potri.015G040400 GGQSYTYKFT VTGQRGTLLW HAHYAWQRAS VYGAFIIYPR ---IPYPFSH PIQAEIPIIF 167
Potri.012G048900 GGQSYTYKFT VIKQRGTLLW HAHYAWQRAS VYGALIIYPR ---MPYPFSA QIQAEIPIIL 167
Potri.016G107900 PGGKYTYKFK LLQQEGTLWW HAHFSLLRAT VYGALIIRPR -SGHPYPFPK -PNKEIPILL 162
Potri.016G106100 PGGKYTYKFK LLQQEGTLWW HAHFSLLRAT VYGALIIRPR -SGHPYPFPK -PNKEIPILL 162
Potri.016G106300 PGGKYTYKFK LLQQEGTLWW HAHFSLLRAT VYGALIIRPR -SGHPYPFPK -PNKEVPILL 162
Potri.016G107500 PGGKYTYKFE LLQQEGTLWW HAHVSFLRAT VYGALVIRPR -SGHPYPFPK -PHREVPILL 162
Potri.016G106000 PGGKYTYKFE LLQQEGTLWW HAHVSFLRAT VYGALVIRPR -SGHPYPFPK -PHREVPILL 162
Potri.006G094100 PGGKYTYQFK LLKQEGTLWW HAHVSWLRAT VYGALIIRPR -SGHPYPFPK -PDKEVPILF 162
Potri.019G121700 PGRSYTYRFT IENQEGTLWW HAHSRWLRAT VYGALIIHPK -LGSPYSFPM -PIREIPILL 172
Potri.019G124300 PGRSYTYRFT IENQEGTLWW HAHSRWLRAT VYGALIIHPK -LGSPYSFPM -PIREIPILL 172
Potri.013G152700 PGRSYTYRFT IENQEGTLWW HAHSRWLRAT VYGALIIHPK -LGSPYPFPM -PRTEIPILL 172
Potri.010G183600 PGGSYTYRFN IEGQEGTLWW HAHSSWLRAT VYGALIIHPR -EGSSYPFAK -PKRETPILL 171
Potri.008G073700 PGGSYTYRFT IEGQEGTLWW HAHSSWLRAT VYGALIIHPR -EGSSYPFSK -PKRETPILL 170
Potri.008G073800 PGGSYTYKFT IQGQEGTLWW HAHSSWLRAT VYGALIVHPK -EGSPYPFSK QPKRETAILL 175
Potri.010G183500 PGGSYTYRFT IQGQEGTLWW HAHSSWLRAT VYGALIIHPK -EGSSYPFSK QPKRETAILL 175
Potri.014G100600 PGQTFTYEFT LVGQKGTFFW HAHVSWLRAT VYGALVVYPK P-GVPYPFKY P-YEEHIVIL 172
Potri.019G088900 PGKNFTYEII LSDEEGTLWW HAHSDWTRAT VHGAIVISPA -RGTTYPFPA P-YAEQTIII 168
Potri.019G088800 PGKNFTYEII LSDEEGTLWW HAHSDWTRAT VHGAIVISPA -RGTTYPFPA P-YAEQTIII 168
Potri.019G088600 PGKNFTYEII LSDEEGTLWW HAHSDWTRAT VHGAIVISPA -RGTTYPFPA P-YAEQTIII 168
Potri.019G088700 PGKNFTYEII LSDEEGTLWW HAHSDWTRAT VHGAIVISPA -RGTTYPFPA P-YAEQTIII 168
Potri.019G088500 PGKNFTYEII LSDEEGTLWW HAHSDWTRAT VHGAIVISPA -RGTTYPFPA P-YAEQTIII 168
Potri.001G206200 PGRRFNYKIT LTTEEGTIWW HAHNSWARAT VHGALIIYPK -HGSHYPFPK P-HAE----F 160
Potri.005G200700 PGGKFKQKII FSTEEGTLWW HAHSDWSRAT VHGPIIVYPK INGTGYPFSK P-LVEVPIIL 165
Potri.011G071100 PGGKFSQRVI FSEEEGTLWW HAHSDWTRAT VYGAIVIYPN -EGTKYPFLA P-HADVPIIL 161
Potri.005G200600 PGGKFSQRVI FSNEEGTLWW HAHSDWTRAT VYGAIVIYPK -KGTEYPFPM P-HADVPIIL 165
Potri.005G200500 PGGKFKQRVI FSDEEGTLWW HAHSDWTRAT VYGAIVIYPK -KGTEYPFPA P-HADVPIIL 165
```

4.3bits
Sequence logo
0.0bits

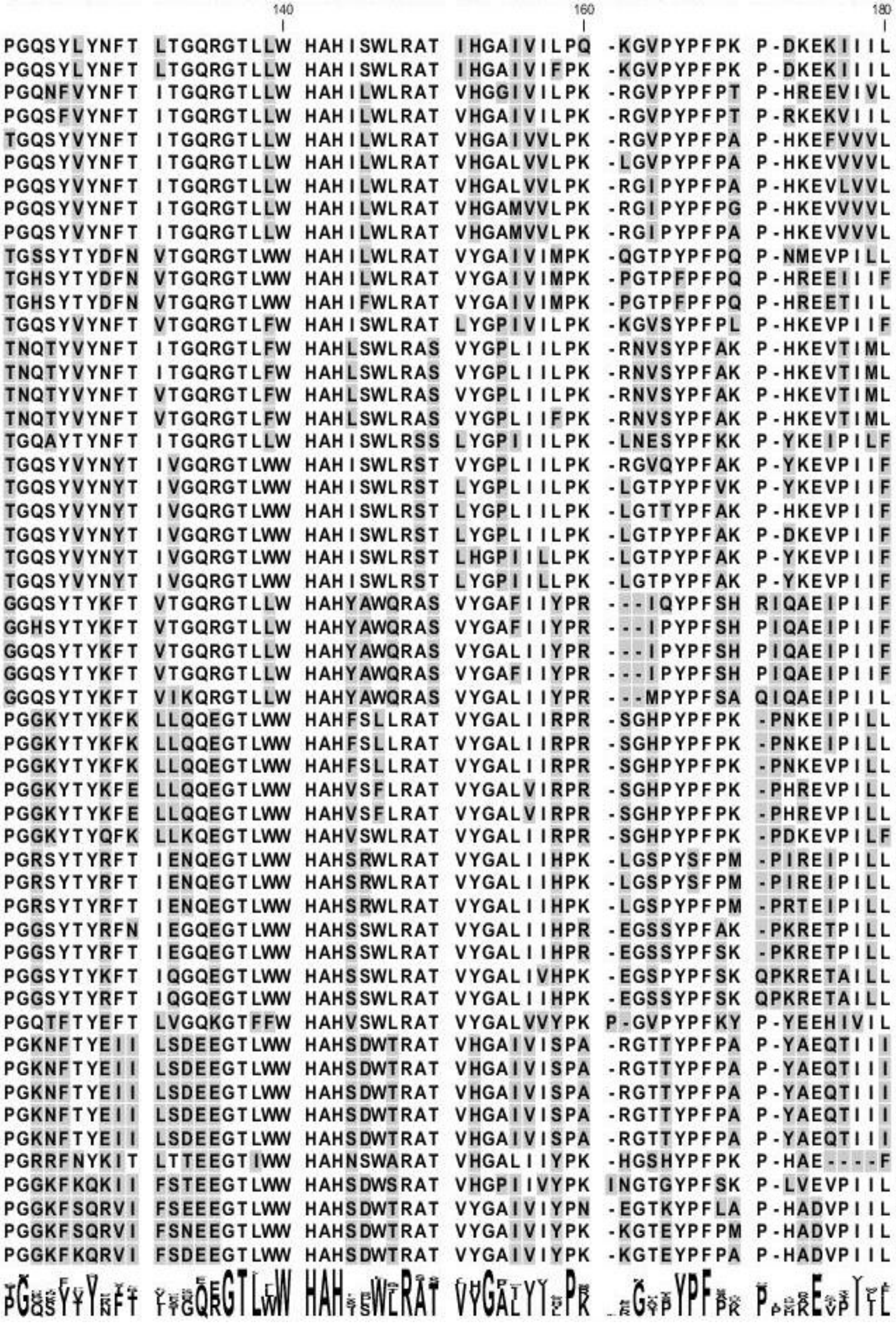

Supplementary Figure S1 (cont.)

		140			160		180	
Potri.008G064000	PGQSYLYNFT	LTGQRGTLLW	HAHISWLRAT	IHGAIVILPQ	-KGVPYPFPK	P-DKEKIIIL		163
Potri.010G193100	PGQSYLYNFT	LTGQRGTLLW	HAHISWLRAT	IHGAIVIFPK	-KGVPYPFPK	P-DKEKIIIL		162
Potri.001G248700	PGQNFVYNFT	ITGQRGTLLW	HAHILWLRAT	VHGGIVILPK	-RGVPYPFPT	P-HREEVIVL		164
Potri.009G042500	PGQSFVYNFT	ITGQRGTLLW	HAHILWLRAT	VHGAIVILPK	-RGVPYPFPT	P-RKEKVIIL		163
Potri.016G112100	TGQSYVYNFT	ITGQRGTLLW	HAHILWLRAT	VHGAIVVLPK	-RGVPYPFPA	P-HKEFVVVL		169
Potri.016G112000	PGQSYVYNFT	ITGQRGTLLW	HAHILWLRAT	VHGALVVLPK	-LGVPYPFPA	P-HKEVVVVL		162
Potri.006G097000	PGQSYVYNFT	ITGQRGTLLW	HAHILWLRAT	VHGALVVLPK	-RGIPYPFPA	P-HKEVLVVL		164
Potri.006G096900	PGQSYVYNFT	ITGQRGTLLW	HAHILWLRAT	VHGAMVVLPK	-RGIPYPFPG	P-HKEVVVVL		163
Potri.006G097100	PGQSYVYNFT	ITGQRGTLLW	HAHILWLRAT	VHGAMVVLPK	-RGIPYPFPA	P-HKEVVVVL		164
Potri.007G023300	TGSSYTYDFN	VTGQRGTLWW	HAHILWLRAT	VYGAIVIMPK	-QGTPYPFPQ	P-NMEVPILL		168
Potri.009G102700	TGHSYTYDFN	VTGQRGTLWW	HAHILWLRAT	VYGAIVIMPK	-PGTPFPFPQ	P-HREEIIIF		167
Potri.004G156400	TGHSYTYDFN	VTGQRGTLWW	HAHIFWLRAT	VYGAIVIMPK	-PGTPFPFPQ	P-HREETIIL		170
Potri.006G087500	TGQSYVYNFT	VTGQRGTLFW	HAHISWLRAT	LYGPIVILPK	-KGVSYPFPL	P-HKEVPIIF		169
Potri.009G156800	TNQTYVYNFT	ITGQRGTLFW	HAHLSWLRAS	VYGPLIILPK	-RNVSYPFAK	P-HKEVTIML		176
Potri.009G156600	TNQTYVYNFT	ITGQRGTLFW	HAHLSWLRAS	VYGPLIILPK	-RNVSYPFAK	P-HKEVTIML		176
Potri.001G184300	TNQTYVYNFT	VTGQRGTLFW	HAHLSWLRAS	VYGPLIILPK	-RNVSYPFAK	P-HKEVTIML		176
Potri.009G034500	TNQTYVYNFT	VTGQRGTLFW	HAHLSWLRAS	VYGPLIIFPK	-RNVSYPFAK	P-HKEVTIML		176
Potri.006G087100	TGQAYTYNFT	ITGQRGTLLW	HAHISWLRSS	LYGPIIILPK	-LNESYPFKK	P-YKEIPILF		173
Potri.001G341600	TGQSYVYNYT	IVGQRGTLWW	HAHISWLRST	VYGPLIILPK	-RGVQYPFAK	P-YKEVPIIF		170
Potri.001G054600	TGQSYVYNYT	IVGQRGTLWW	HAHISWLRST	LYGPLIILPK	-LGTPYPFVK	P-YKEVPIIF		171
Potri.001G401300	TGQSYVYNYT	IVGQRGTLWW	HAHISWLRST	LYGPLIILPK	-LGTTYPFAK	P-HKEVPIIF		171
Potri.001G401100	TGQSYVYNYT	IVGQRGTLWW	HAHISWLRST	LYGPLIILPK	-LGTPYPFAK	P-DKEVPIIF		171
Potri.011G120300	TGQSYVYNYT	IVGQRGTLWW	HAHISWLRST	LHGPIILLPK	-LGTPYPFAK	P-YKEVPIIF		171
Potri.011G120200	TGQSYVYNYT	IVGQRGTLWW	HAHISWLRST	LYGPIILLPK	-LGTPYPFAK	P-YKEVPIIF		171
Potri.015G040700	GGQSYTYKFT	VTGQRGTLLW	HAHYAWQRAS	VYGAFIIYPR	---IQYPFSH	RIQAEIPIIF		150
Potri.015G040800	GGHSYTYKFT	VTGQRGTLLW	HAHYAWQRAS	VYGAFIIYPR	---IPYPFSH	PIQAEIPIIF		68
Potri.015G040600	GGQSYTYKFT	VTGQRGTLLW	HAHYAWQRAS	VYGALIIYPR	---IPYPFSH	PIQAEIPIIF		167
Potri.015G040400	GGQSYTYKFT	VTGQRGTLLW	HAHYAWQRAS	VYGAFIIYPR	---IPYPFSH	PIQAEIPIIF		167
Potri.012G048900	GGQSYTYKFT	VIKQRGTLLW	HAHYAWQRAS	VYGALIIYPR	---MPYPFSA	QIQAEIPIIL		167
Potri.016G107900	PGGKYTYKFK	LLQQEGTLWW	HAHFSLLRAT	VYGALIIRPR	-SGHPYPFPK	-PNKEIPILL		162
Potri.016G106100	PGGKYTYKFK	LLQQEGTLWW	HAHFSLLRAT	VYGALIIRPR	-SGHPYPFPK	-PNKEIPILL		162
Potri.016G106300	PGGKYTYKFK	LLQQEGTLWW	HAHFSLLRAT	VYGALIIRPR	-SGHPYPFPK	-PNKEVPILL		162
Potri.016G107500	PGGKYTYKFE	LLQQEGTLWW	HAHVSFLRAT	VYGALVIRPR	-SGHPYPFPK	-PHREVPILL		162
Potri.016G106000	PGGKYTYKFE	LLQQEGTLWW	HAHVSFLRAT	VYGALVIRPR	-SGHPYPFPK	-PHREVPILL		162
Potri.006G094100	PGGKYTYQFK	LLKQEGTLWW	HAHVSWLRAT	VYGALIIRPR	-SGHPYPFPK	-PDKEVPILF		162
Potri.019G121700	PGRSYTYRFT	IENQEGTLWW	HAHSRWLRAT	VYGALIIHPK	-LGSPYSFPM	-PIREIPILL		172
Potri.019G124300	PGRSYTYRFT	IENQEGTLWW	HAHSRWLRAT	VYGALIIHPK	-LGSPYSFPM	-PIREIPILL		172
Potri.013G152700	PGRSYTYRFT	IENQEGTLWW	HAHSRWLRAT	VYGALIIHPK	-LGSPYPFPM	-PRTEIPILL		172
Potri.010G183600	PGGSYTYRFN	IEGQEGTLWW	HAHSSWLRAT	VYGALIIHPR	-EGSSYPFAK	-PKRETPILL		171
Potri.008G073700	PGGSYTYRFT	IEGQEGTLWW	HAHSSWLRAT	VYGALIIHPR	-EGSSYPFSK	-PKRETPILL		170
Potri.008G073800	PGGSYTYKFT	IQGQEGTLWW	HAHSSWLRAT	VYGALIVHPK	-EGSPYPFSK	QPKRETAILL		175
Potri.010G183500	PGGSYTYRFT	IQGQEGTLWW	HAHSSWLRAT	VYGALIIHPK	-EGSSYPFSK	QPKRETAILL		175
Potri.014G100600	PGQTFTYEFT	LVGQKGTFFW	HAHVSWLRAT	VYGALVVYPK	P-GVPYPFKY	P-YEEHIVIL		172
Potri.019G088900	PGKNFTYEII	LSDEEGTLWW	HAHSDWTRAT	VHGAIVISPA	-RGTTYPFPA	P-YAEQTIII		168
Potri.019G088800	PGKNFTYEII	LSDEEGTLWW	HAHSDWTRAT	VHGAIVISPA	-RGTTYPFPA	P-YAEQTIII		168
Potri.019G088600	PGKNFTYEII	LSDEEGTLWW	HAHSDWTRAT	VHGAIVISPA	-RGTTYPFPA	P-YAEQTIII		168
Potri.019G088700	PGKNFTYEII	LSDEEGTLWW	HAHSDWTRAT	VHGAIVISPA	-RGTTYPFPA	P-YAEQTIII		168
Potri.019G088500	PGKNFTYEII	LSDEEGTLWW	HAHSDWTRAT	VHGAIVISPA	-RGTTYPFPA	P-YAEQTIII		168
Potri.001G206200	PGRRFNYKIT	LTTEEGTIWW	HAHNSWARAT	VHGALIIYPK	-HGSHYPFPK	P-HAE----F		160
Potri.005G200700	PGGKFKQKII	FSTEEGTLWW	HAHSDWSRAT	VHGPIIVYPK	INGTGYPFSK	P-LVEVPIIL		165
Potri.011G071100	PGGKFSQRVI	FSEEEGTLWW	HAHSDWTRAT	VYGAIVIYPN	-EGTKYPFLA	P-HADVPIIL		161
Potri.005G200600	PGGKFSQRVI	FSNEEGTLWW	HAHSDWTRAT	VYGAIVIYPK	-KGTEYPFPM	P-HADVPIIL		165
Potri.005G200500	PGGKFKQRVI	FSDEEGTLWW	HAHSDWTRAT	VYGAIVIYPK	-KGTEYPFPA	P-HADVPIIL		165
Sequence logo (4.3bits / 0.0bits)	[illegible]	[illegible]	[illegible]	[illegible]	[illegible]	[illegible]		

Supplementary Figure S1 (cont.)

200 220 240

Potri.008G064000	GEWWKADVEA	VVNQATQTGL	PPNISDAHIV	NGQTGAVPGC	PSPG-FTLHV	ESGKTYLLRI	222
Potri.010G193100	SEWWKADVEA	VVNQATMTGL	PPNISDAHTV	NGHTGAVPGC	TSPG-FTLHV	ESGKTYLLRI	221
Potri.001G248700	GEWWKSDVEA	VINEAMNSGR	APNVSDAHTI	NGHPGPVSGC	SSQGGYNLPV	RPGKTYMLRI	224
Potri.009G042500	GEWWKSDVEA	VINEATKSGI	APNVSDAHTI	NGHPGPVSAC	SSHGGYNLSV	HPGKTYMLRI	223
Potri.016G112100	AEWWKSDTEA	VINEALKSGL	APNVSDAHTI	NGHPGAVSAC	PSQGGFTLPV	ESGKTYMLRL	229
Potri.016G112000	AEWWKSDTEA	VINEALKSGL	APNVSDAHTI	NGHPGAVSTC	SSQGGFTLPV	QSGKTYMLRL	222
Potri.006G097000	AEWWKSDTEA	VINEALKSGL	APNVSDAHTI	NGHPGAVSAC	SSQGGFTLPV	KSGETYMLRL	224
Potri.006G096900	AEWWKSDTEA	VINEALKSGL	APNVSDAHTI	NGHPGAVSTC	SSQGGFTLPV	KSGETYMLRL	223
Potri.006G097100	AEWWKSDTEA	VINEALKSGL	APNVSDAHTI	NGHPGAVSAC	SSQGGFTLPV	KSGETYMLRL	224
Potri.007G023300	GEWWNTDVEE	VEKQGTEMGL	PPNMSDAHTI	NGKPGPLFPC	SEKHTFAMEI	ESGKTYLLRI	228
Potri.009G102700	GEWWNNDVED	IEKQGNKLGL	PPNASDAHTI	NGKPGPLFPC	SEKHTFTLEV	EQAKTYLLRI	227
Potri.004G156400	GEWWNNDVEE	IEKQGSKLGL	PPNASDAHTI	NGKPGTLFPC	SEKHTFAMEV	EQGKTYLLRI	230
Potri.006G087500	GEWWKADTEK	IISQALKTGG	APNISDAYTI	NGHPGLLYNC	SAKDTFKLKV	KPGKTYLLRL	229
Potri.009G156800	GEWFNADTEA	VISQALQTGG	GPNVSEAYTF	NGLPGPLYNC	SENNTYKLKV	KPGKTYLLRL	236
Potri.009G156600	GEWFNADTEA	VISQALQTGG	GPNVSEAYTF	NGLPGPLYNC	SENNTYKLKV	KPGKTYLLRL	236
Potri.001G184300	GEWFNADTEA	VISQALQTGG	GPNVSEAYTF	NGLPGPLYNC	SENNTYKLKV	KPGKTYLLRL	236
Potri.009G034500	GEWFNADPEA	VIRQALQTGG	GPNVSEAYTF	NGLTGPLYNC	SANNTYKLKV	KPGKTYLLRL	236
Potri.006G087100	GEWFNVDPEA	VIAQALQTGA	GPNVSDAYTI	NGLPGPLYNC	SAKDTYKLKV	KPGKTYLLRL	233
Potri.001G341600	GEWFNVDPEA	VISQALQTGG	GPNVSDAYTI	NGLPGPLYNC	SAEDTFKLKV	KPGKTYMLRL	230
Potri.001G054600	GEWFNADPEA	IINQALQTGG	GPNVSDAYTI	NGLPGPLYNC	SAKDTFKLKV	KPGKTYLLRL	231
Potri.001G401300	GEWFNADPEA	IINQAMQTGG	GPNVSDAYTI	NGFPGPLYNC	SAKDTFKLKV	KPGKTYLLRM	231
Potri.001G401100	GEWFNADPEA	IINQAMQTGG	GPNVSDAYTI	NGLPGPLYNC	SAKNTFKLKV	KPGKTYLLRL	231
Potri.011G120300	GEWFNADPEA	IISQAMQTGG	GPNVSDAYTI	NGLPGPLYNC	SAKDTFKLKV	KPGKTYLLRM	231
Potri.011G120200	GEWFNADPEA	IINQAMQTGG	GPNVSDAYTI	NGLPGPLYNC	SAKDTFKLKV	KPGKTYLLRM	231
Potri.015G040700	GEWWNGDPDE	VEKTMLLTGG	GPDSSNAYTI	NGLPGPLYPC	SNQDTFIQTV	EYGKTYLLRI	210
Potri.015G040800	GEWWNGDPDE	VEKTMLLTGG	GPDSSNAYTI	NGLPGPLYPC	SNQDTFIQTV	EYGKTYLLRI	128
Potri.015G040600	GEWWNGDPDE	IEKTMLLTGG	GPDSSNAYTI	NGLPGPLYPC	SNQDTFIQTV	EYGKTYLLRI	227
Potri.015G040400	GEWWNGDPDE	VENRTMLTGA	GPDSSNAYTI	NGLPGPLYPC	SNQDTYIQTV	EYGKTYMLRI	227
Potri.012G048900	GEWWNGDPDE	VEKIMMLTGA	GPDSSNAYTI	NGMPGPLYPC	SNRDTFIQTV	EYGRTYMLRI	227
Potri.016G107900	GEWWNADVVG	IEREAAATGA	PPKISDAYTI	NGLPGDLYNC	SQNRMYKLKV	QKGKTYLLRI	222
Potri.016G106100	GEWWNADVVG	IERKAAATGA	PPKISDAYTI	NGLPGDLYNC	SQNRMYKLKV	QKGKTYLLRI	222
Potri.016G106300	GEWWNGDVVG	IERKAAATGA	SPKISDAYTI	NGLPGDLYNC	SQDRMYKLKV	QKGKTYLLRI	222
Potri.016G107500	GEWWNANVVD	VENQAEAIGA	PPNISDAYTI	NGLPGDLYNC	SQNRMYKLKV	QKGKTYLLRI	222
Potri.016G106000	GEWWNANVVD	VENQAEAIGA	PPNISDAYTI	NGLPGDLYNC	SQNRMYKLKV	QKGKTYLLRI	222
Potri.006G094100	GEWWNANVVD	VENQALASGA	APNTSDAFTI	NGLPGDLYPC	SQNRIFKLKV	QKGKTYLLRI	222
Potri.019G121700	GEWWVRNPMD	VLRLADFTGA	APNVSDAYTI	NGQPGDLYRC	SKQETVRFPV	DPGETILLRV	232
Potri.019G124300	GEWWVRNPMD	VLRLADFTGA	APNVSDAYTI	NGQPGDLYRC	SKQETVRFPV	DPGETILLRV	232
Potri.013G152700	GEWWDRNPMD	VLRIADFTGA	APNISDAYTI	NGQPGDLYRC	SKQETVRFPV	GSGETILLRV	232
Potri.010G183600	GEWWDANPVD	VVREATRTGA	APNISDAYTI	NGQPGDLYNC	SSEDTTIVPI	ASGETNLLRV	231
Potri.008G073700	GEWWDTNPID	VVREATRTGA	APNISDAYTI	NGQPGDLFNC	SSKDTTIVPI	DSGETNLLRV	230
Potri.008G073800	GEWWNANPID	VVREATRTGG	APNVSDAYTV	NGQPGDLYNC	SSQDTVIVPI	DSGETNLLRV	235
Potri.010G183500	GEWWNANPID	VVRESTRTGG	TPNSSDAYTI	NGQPGDLYNC	SSQDTVIVPI	DSGETNLLRV	235
Potri.014G100600	GEYWLQDIVH	LERQVVASGG	GPPPANAYTI	NGHPGPNYNC	SATDVYKIDV	LPGKTYLLRL	232
Potri.019G088900	GSWFKGDVKA	VIDEALATGA	GPAISNSLTI	NGQPGDLYPC	SEENTYRLKV	NSGRTYLLRV	228
Potri.019G088800	GSWFKRDVKA	VIDEVLATGV	GPAPSNSLTI	NGQPGDLYPC	SEENTYRLKV	NSGRTYLLRV	228
Potri.019G088600	GSWFKGDVKA	VIDEALATGG	GPNISNSLTI	NGQPGDLYPC	SEENTYRLKV	NSGRTYLLRV	228
Potri.019G088700	GSWFKGDVKA	VIDDALATGG	GPAISNSLTI	NGQPGDKYPC	SEENTYRLMV	NSGRTYLLRV	228
Potri.019G088500	GSWFKGDVKA	VIDEALATGV	GPNISNSLTI	NGQPGDLYPC	SDKNTYRLKV	NSGRTYLLRV	228
Potri.001G206200	GEWWKKDVMK	IPGDANITGG	EPTLSAAFTI	NGEPGYMYPC	SKAGTFKMMV	EQGKTYLLRI	220
Potri.005G200700	GEWWKRDVMD	VLQEAVITGG	DPAVSDAFTI	NGQPGDLYPC	SKSETIKLNV	HQGNSYLLRI	225
Potri.011G071100	GEWWKKDIFD	IFDQFRASGA	DPNVSDAYTI	NGQPGDLYPC	SKSDTFKLSV	DYGKTYLLRL	221
Potri.005G200600	GEWWKKDIFE	IFDQFRASGA	DPDVSDAYTI	NGQPGDLYPC	SKSDTFKLSV	DYGKTYLLRL	225
Potri.005G200500	GEWWKKDIFE	IFDQFRASGA	DPNVSDSYTI	NGQPGDLYPC	SKSDTFKLSV	DYGKTYLLRL	225

4.3bits
Sequence logo
0.0bits

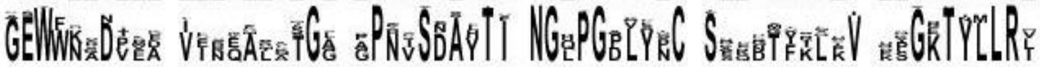

Supplementary Figure S1 (cont.)

		200		220		240	
Potri.008G064000	GEWWKADVEA	VVNQATQTGL	PPNISDAHIV	NGQTGAVPGC	PSPG-FTLHV	ESGKTYLLRI	222
Potri.010G193100	SEWWKADVEA	VVNQATMTGL	PPNISDAHTV	NGHTGAVPGC	TSPG-FTLHV	ESGKTYLLRI	221
Potri.001G248700	GEWWKSDVEA	VINEAMNSGR	APNVSDAHTI	NGHPGPVSGC	SSQGGYNLPV	RPGKTYMLRI	224
Potri.009G042500	GEWWKSDVEA	VINEATKSGI	APNVSDAHTI	NGHPGPVSAC	SSHGGYNLSV	HPGKTYMLRI	223
Potri.016G112100	AEWWKSDTEA	VINEALKSGL	APNVSDAHTI	NGHPGAVSAC	PSQGGFTLPV	ESGKTYMLRL	229
Potri.016G112000	AEWWKSDTEA	VINEALKSGL	APNVSDAHTI	NGHPGAVSTC	SSQGGFTLPV	QSGKTYMLRL	222
Potri.006G097000	AEWWKSDTEA	VINEALKSGL	APNVSDAHTI	NGHPGAVSAC	SSQGGFTLPV	KSGETYMLRL	224
Potri.006G096900	AEWWKSDTEA	VINEALKSGL	APNVSDAHTI	NGHPGAVSTC	SSQGGFTLPV	KSGETYMLRL	223
Potri.006G097100	AEWWKSDTEA	VINEALKSGL	APNVSDAHTI	NGHPGAVSAC	SSQGGFTLPV	KSGETYMLRL	224
Potri.007G023300	GEWWNTDVEE	VEKQGTEMGL	PPNMSDAHTI	NGKPGPLFPC	SEKHTFAMEI	ESGKTYLLRI	228
Potri.009G102700	GEWWNNDVED	IEKQGNKLGL	PPNASDAHTI	NGKPGPLFPC	SEKHTFTLEV	EQAKTYLLRI	227
Potri.004G156400	GEWWNNDVEE	IEKQGSKLGL	PPNASDAHTI	NGKPGTLFPC	SEKHTFAMEV	EQGKTYLLRI	230
Potri.006G087500	GEWWKADTEK	IISQALKTGG	APNISDAYTI	NGHPGLLYNC	SAKDTFKLKV	KPGKTYLLRL	229
Potri.009G156800	GEWFNADTEA	VISQALQTGG	GPNVSEAYTF	NGLPGPLYNC	SENNTYKLKV	KPGKTYLLRL	236
Potri.009G156600	GEWFNADTEA	VISQALQTGG	GPNVSEAYTF	NGLPGPLYNC	SENNTYKLKV	KPGKTYLLRL	236
Potri.001G184300	GEWFNADTEA	VISQALQTGG	GPNVSEAYTF	NGLPGPLYNC	SENNTYKLKV	KPGKTYLLRL	236
Potri.009G034500	GEWFNADPEA	VIRQALQTGG	GPNVSEAYTF	NGLTGPLYNC	SANNTYKLKV	KPGKTYLLRL	236
Potri.006G087100	GEWFNVDPEA	VIAQALQTGA	GPNVSDAYTI	NGLPGPLYNC	SAKDTYKLKV	KPGKTYLLRL	233
Potri.001G341600	GEWFNVDPEA	VISQALQTGG	GPNVSDAYTI	NGLPGPLYNC	SAEDTFKLKV	KPGKTYMLRL	230
Potri.001G054600	GEWFNADPEA	IINQALQTGG	GPNVSDAYTI	NGLPGPLYNC	SAKDTFKLKV	KPGKTYLLRL	231
Potri.001G401300	GEWFNADPEA	IINQAMQTGG	GPNVSDAYTI	NGFPGPLYNC	SAKDTFKLKV	KPGKTYLLRM	231
Potri.001G401100	GEWFNADPEA	IINQAMQTGG	GPNVSDAYTI	NGLPGPLYNC	SAKNTFKLKV	KPGKTYLLRL	231
Potri.011G120300	GEWFNADPEA	IISQAMQTGG	GPNVSDAYTI	NGLPGPLYNC	SAKDTFKLKV	KPGKTYLLRM	231
Potri.011G120200	GEWFNADPEA	IINQAMQTGG	GPNVSDAYTI	NGLPGPLYNC	SAKDTFKLKV	KPGKTYLLRM	231
Potri.015G040700	GEWWNGDPDE	VEKTMLLTGG	GPDSSNAYTI	NGLPGPLYPC	SNQDTFIQTV	EYGKTYLLRI	210
Potri.015G040800	GEWWNGDPDE	VEKTMLLTGG	GPDSSNAYTI	NGLPGPLYPC	SNQDTFIQTV	EYGKTYLLRI	128
Potri.015G040600	GEWWNGDPDE	IEKTMLLTGG	GPDSSNAYTI	NGLPGPLYPC	SNQDTFIQTV	EYGKTYLLRI	227
Potri.015G040400	GEWWNGDPDE	VENRTMLTGA	GPDSSNAYTI	NGLPGPLYPC	SNQDTYIQTV	EYGKTYMLRI	227
Potri.012G048900	GEWWNGDPDE	VEKIMMLTGA	GPDSSNAYTI	NGMPGPLYPC	SNRDTFIQTV	EYGRTYMLRI	227
Potri.016G107900	GEWWNADVVG	IEREAAATGA	PPKISDAYTI	NGLPGDLYNC	SQNRMYKLKV	QKGKTYLLRI	222
Potri.016G106100	GEWWNADVVG	IERKAAATGA	PPKISDAYTI	NGLPGDLYNC	SQNRMYKLKV	QKGKTYLLRI	222
Potri.016G106300	GEWWNGDVVG	IERKAAATGA	SPKISDAYTI	NGLPGDLYNC	SQDRMYKLKV	QKGKTYLLRI	222
Potri.016G107500	GEWWNANVVD	VENQAEAIGA	PPNISDAYTI	NGLPGDLYNC	SQNRMYKLKV	QKGKTYLLRI	222
Potri.016G106000	GEWWNANVVD	VENQAEAIGA	PPNISDAYTI	NGLPGDLYNC	SQNRMYKLKV	QKGKTYLLRI	222
Potri.006G094100	GEWWNANVVD	VENQALASGA	APNTSDAFTI	NGLPGDLYPC	SQNRIFKLKV	QKGKTYLLRI	222
Potri.019G121700	GEWWVRNPMD	VLRLADFTGA	APNVSDAYTI	NGQPGDLYRC	SKQETVRFPV	DPGETILLRV	232
Potri.019G124300	GEWWVRNPMD	VLRLADFTGA	APNVSDAYTI	NGQPGDLYRC	SKQETVRFPV	DPGETILLRV	232
Potri.013G152700	GEWWDRNPMD	VLRIADFTGA	APNISDAYTI	NGQPGDLYRC	SKQETVRFPV	GSGETILLRV	232
Potri.010G183600	GEWWDANPVD	VVREATRTGA	APNISDAYTI	NGQPGDLYNC	SSEDTTIVPI	ASGETNLLRV	231
Potri.008G073700	GEWWDTNPID	VVREATRTGA	APNISDAYTI	NGQPGDLFNC	SSKDTTIVPI	DSGETNLLRV	230
Potri.008G073800	GEWWNANPID	VVREATRTGG	APNVSDAYTV	NGQPGDLYNC	SSQDTVIVPI	DSGETNLLRV	235
Potri.010G183500	GEWWNANPID	VVRESTRTGG	TPNSSDAYTI	NGQPGDLYNC	SSQDTVIVPI	DSGETNLLRV	235
Potri.014G100600	GEYWLQDIVH	LERQVVASGG	GPPPANAYTI	NGHPGPNYNC	SATDVYKIDV	LPGKTYLLRL	232
Potri.019G088900	GSWFKGDVKA	VIDEALATGA	GPAISNSLTI	NGQPGDLYPC	SEENTYRLKV	NSGRTYLLRV	228
Potri.019G088800	GSWFKRDVKA	VIDEVLATGV	GPAPSNSLTI	NGQPGDLYPC	SEENTYRLKV	NSGRTYLLRV	228
Potri.019G088600	GSWFKGDVKA	VIDEALATGG	GPNISNSLTI	NGQPGDLYPC	SEENTYRLKV	NSGRTYLLRV	228
Potri.019G088700	GSWFKGDVKA	VIDDALATGG	GPAISNSLTI	NGQPGDKYPC	SEENTYRLMV	NSGRTYLLRV	228
Potri.019G088500	GSWFKGDVKA	VIDEALATGV	GPNISNSLTI	NGQPGDLYPC	SDKNTYRLKV	NSGRTYLLRV	228
Potri.001G206200	GEWWKKDVMK	IPGDANITGG	EPTLSAAFTI	NGEPGYMYPC	SKAGTFKMMV	EQGKTYLLRI	220
Potri.005G200700	GEWWKRDVMD	VLQEAVITGG	DPAVSDAFTI	NGQPGDLYPC	SKSETIKLNV	HQGNSYLLRI	225
Potri.011G071100	GEWWKKDIFD	IFDQFRASGA	DPNVSDAYTI	NGQPGDLYPC	SKSDTFKLSV	DYGKTYLLRL	221
Potri.005G200600	GEWWKKDIFE	IFDQFRASGA	DPDVSDAYTI	NGQPGDLYPC	SKSDTFKLSV	DYGKTYLLRL	225
Potri.005G200500	GEWWKKDIFE	IFDQFRASGA	DPNVSDSYTI	NGQPGDLYPC	SKSDTFKLSV	DYGKTYLLRL	225

Sequence logo 4.3bits 0.0bits

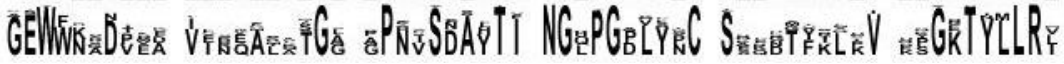

Supplementary Figure S1 (cont.)

```
                                          260                                280                                300
                                            |                                  |                                  |
Potri.008G064000  INAALNDELF FKIAGHNITV VEVDAAYTKP FSTDTIFIGP GQTTNALLTA DKS---VGKY 279
Potri.010G193100  INAALNDELF FKIAGHNITV VEVDATFTKP FSTDTIFIGP GQTTNALLTA DKS---IGKY 278
Potri.001G248700  INAALNEELF FKIAGHQLTV VEVDATYVKP FKIDTIVIAP GQTTNVLVTA NRG---SGKY 281
Potri.009G042500  INAALNEELF FKIAGHQLTV VEVDATYVKP FKIDTVVIAP GQTTNVLVTA NRG---SGQY 280
Potri.016G112100  INAALNEELF FKIAGHKLTL VEVDATYVKP FKTDTVLIAP GQTTNVLVTT NKN---TGKY 286
Potri.016G112000  INAALNEELF FKIAGHKLTV VEVDATYVKP FKTDTVLIAP GQTTNVLVTT NKN---TGKY 279
Potri.006G097000  INAALNEELF FKIAGHKLTV VEVDATYVKP FKTDTVLIAP GQTTNVLVTA NKN---TGKY 281
Potri.006G096900  INAALNEELF FKIAGHKLTV VEVDATYVKP FKTDTVLIAP GQTTNVLVTT NKN---TGKY 280
Potri.006G097100  INAALNEELF FKIAGHKLTV VEVDATYVKP FKTDTVLIAP GQTTNVLVTT NKN---TGKY 281
Potri.007G023300  INAALNDELF FGIAGHNMTV VEVDAVYTKP FTTQTILIAP GQTTNVLVLA NQV---PGRY 285
Potri.009G102700  INAALNDELF FAIAGHNMTV VEIDAVYTKP FTTQTILIAP GQTTNVLVQA TQT---PNRY 284
Potri.004G156400  INAALNDELF FAIAGHNMTV VEVDAVYTKH FTTQAVLIAP GQTTNVLVQA TQS---PNRY 287
Potri.006G087500  INAALNDELF FSIANHSLTV VEADAVYVKP FKTHIVLITP GQTTNVLLMA KAKAP-NSTF 288
Potri.009G156800  INAALNDDLF FSIANHTFTV VEVDATYAKP FETNLLVITA GQTTNVLLKA KPIAP-NASF 295
Potri.009G156600  INVALNDDLF FSIANHTFTV VEVDATYAKP FETNLLVITA GQTTNVLLKT KPIAP-NASF 295
Potri.001G184300  INAALNDDLF FSIANHTFTV VEVDATYAKP FETNLLVITA GQTTNVLLKT KSIAP-NASF 295
Potri.009G034500  INAALNDELF FSIANHTFTV VEVDATYVKP FETNLLVITP GQTTNVLLKT KPIAP-NASF 295
Potri.006G087100  INAALNDELF FSIANHTLTV VEADAVYVKP FEADTLLISP GQTTNVLLKT KPHLP-NATF 292
Potri.001G341600  INAALNDELF FSIANHSVTI VDVDAVYVKP FDTETLLITP GQTTNVLLKT KPYFP-NATF 289
Potri.001G054600  INAALNDELF FSIANHTFTV VEADAVYVKP FDTKTLLIAP GQTTNVLLKT KPHHP-NAKF 290
Potri.001G401300  INAALNDELF FSIANHTLTV VDVDAIYVKP FDTETLLIAP GQTTNVLLKT KPHHP-NASF 290
Potri.001G401100  INAALNEELF FSIANHTLTV VGVDAIYVKP FDTETLLIAS GQTTDVLLKT KPHHP-DAKF 290
Potri.011G120300  INAALNDELF FSIANHTVTV VDVDAVYVKP FDAETLLITP GQTTNVLLKT KPDYP-NAQF 290
Potri.011G120200  INAALNDELF FSIANHTVTV VDVDAVYVKP FDAETLLITP GQTTNVLLKT KPDYP-NAQF 290
Potri.015G040700  INAALTNELF FAIAKHTLTV VEVVAVYTKP FATTSIMISP GQTTTVLMTA NKVPDFTGMF 270
Potri.015G040800  INAALTNELF FAIAKHTLTV VEVVAVYTKP FATTSIMISP GQTTTVLMTA NKVPDFTGMF 188
Potri.015G040600  INAALTNELF FAIAKHTLTV VEVDAVYTKP FATTSIMIAP GQTTTVLMTA NQVPDFTGMF 287
Potri.015G040400  INAALADELF FAIAKHTLTV VEVDAVYTKP FATTSIMIAP GQTTTVLMTA NQVPDFTGMF 287
Potri.012G048900  INAALANELF FAIAKHKLTV VEVDAVYTKP FTTTSIMIAP GQTTTVLMTA NQVPDSTGMF 287
Potri.016G107900  INAALDNQLF FKIANHNMTV VAVDAGYTVP YVTDVVVTGP GQTVDVLLAA DQE---VGSY 279
Potri.016G106100  INAALDNQLF FKIANHNMTV VAVDAGYTVP YVTDVVVTGP GQTVDVLLAA DQE---VGSY 279
Potri.016G106300  INAALDNQLF FMIANHNMTV VAVDAGYTVP YVTDVVVTGP GQTVDVLLAA DQE---VGSY 279
Potri.016G107500  INAALNNQLF FKIANHNMTV VAVDAGYTVP YVTDVVVTGP GQTVDVLLAA DQE---VGSY 279
Potri.016G106000  INAALNNQLF FKIANHNMTV VAVDAGYTVP YVTDVVVTGP GQTVDVLLAA DQE---VGSY 279
Potri.006G094100  INAALNNELF FKIANHNMKV VAVDAGYTVP YVTGVVVIGP GQTVDVLLAA DQE---VGSY 279
Potri.019G121700  INSAMNQELF FAVANHILTV VAVDAACTMP FATSFIMIAP GQTTNVLLTA DQT---PGHY 289
Potri.019G124300  INSAMNQELF FAVANHILTV VAVDAACTMP FATSFIMIAP GQTTNVLLTA DQT---PGHY 289
Potri.013G152700  INSALNQELF FGVANHILTV VAVDAAYTKP FTTSVIMIAP GQTTDVLLTA DQT---PGHY 289
Potri.010G183600  INAALNQPLF FTIANHKFTV IGADASYLKP FTTSVIMLGP GQTTDVLISG DQL---PGRY 288
Potri.008G073700  INAALNQPLF FTIANHKFTV VGADASYLKP FTTSVIMLGP GQTTDVLISG DQL---PGRY 287
Potri.008G073800 VNSALNQPLF FTVANHKFTV VGADASYVKP FTTSVLMLGP GQTTDVLISG DQT---PSRY 292
Potri.010G183500 VNSALNQPLF FTVANHKLTV VGADASYVKP FTTSVLMLGP GQTTDVLISG DQN---PSRY 292
Potri.014G100600  INAGLNMENF FAIANHKLTI VEADAEYTKP FTTDRVMLGP GQTMIVLVTA DQT---IGKY 289
Potri.019G088900  INAVMNEEQF FGIAGHSLTV VGQDAAYIKP ITTNYIMITP GQTMDILVTA NQ---PRSYY 285
Potri.019G088800  INAVMNEEQF FGIAGHSLTV VGQDAAYIKP ITTNYIMITP GQTMDILVTA NQ---PPSYY 285
Potri.019G088600  INAVMNEEQF FGIAGHSLTV VGQDAAYIKP ITTNYIMITP GQTMDILVTA NR---PRSYY 285
Potri.019G088700  INAVMNEEQF FGIAGHSLTV VGQDAAYIKP ITTNYIMITP GQTMDILVTA NR---PRSYY 285
Potri.019G088500  INAVMNEEQF FGIAGHSLTV VGQDAAYIKP ITTNYIMITP GQTMEILVTA NQ---PPSYY 285
Potri.001G206200  INAVLDENLF FSIAKHKLTI VGKDGCYLKP FTSDYLMITP GQTMDVLFEA NQ---PPSHY 277
Potri.005G200700 VNAALNTILF FSVAKHNLTV VGIDGSYAKQ LTSGYITIAS GQTIDAVLHA NQ---DPNHY 282
Potri.011G071100  INAALQDILF FSITDHQVTV VGTDAGYTKP LKVDYVAISP GQTIDVLLEA NQ---PLDHY 278
Potri.005G200600  INAALQDILF FSITNHQVTV VGTDASYTKP LKVDYIAISP GQTIDVLLEA NQ---PLDHY 282
Potri.005G200500  INAALQDIVF FSITNHQVTV VGTDASYTKP LKVDYIAISP GQTIDVLLEA NQ---PLDHY 282
```

4.3bits

Sequence logo

0.0bits

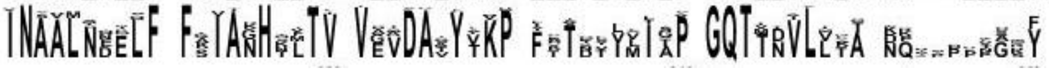

Supplementary Figure S1 (cont.)

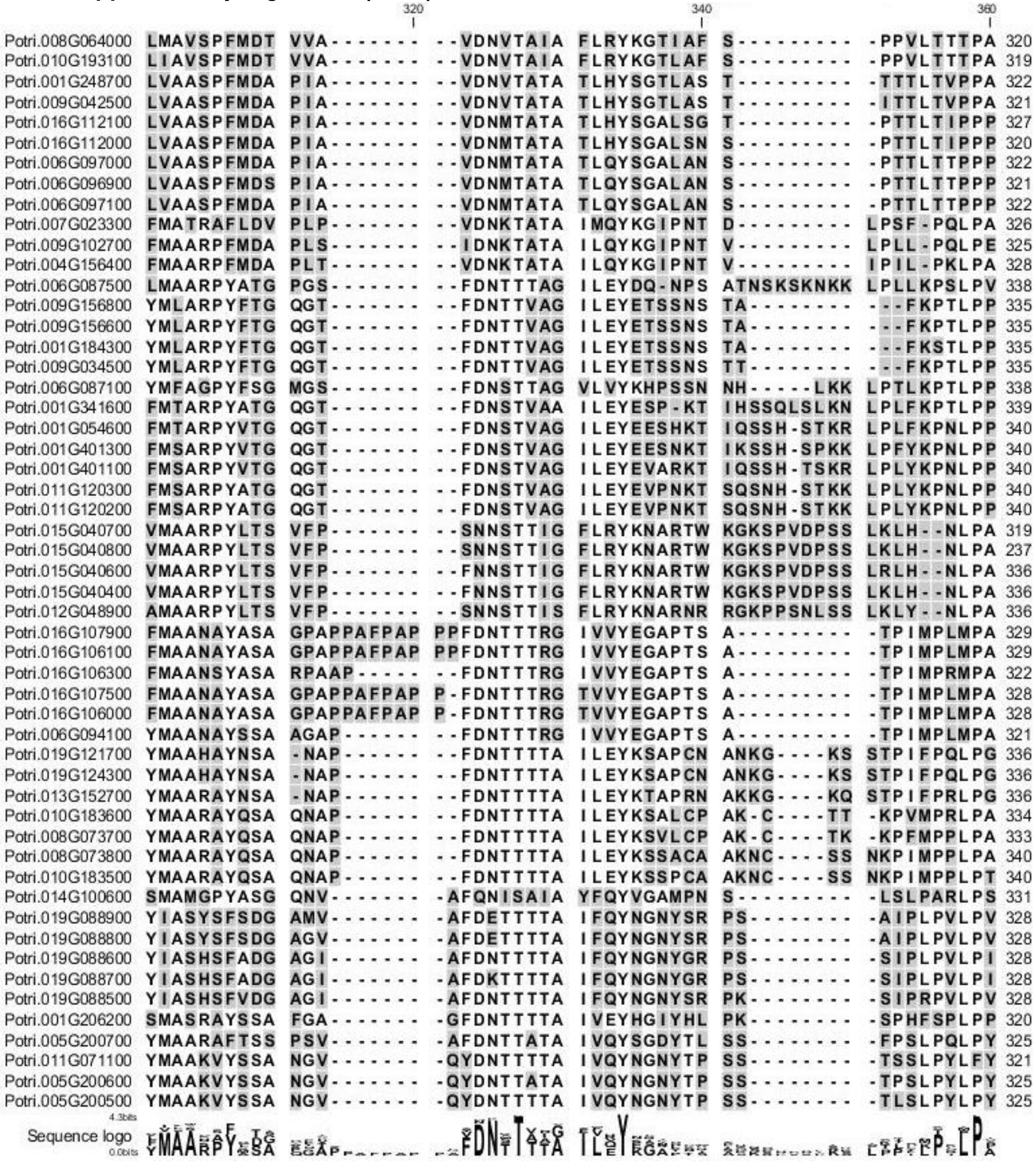

Supplementary Figure S1 (cont.)

```
                                     380                                        400                                        420
Potri.008G064000  INATPVTSTF  MDNLRSLNSK  KFPA--NVPL  TVDHSLYFTI  GVGIDPCA--  -T----CV--  369
Potri.010G193100  INATPATSTF  MDKLRSLNSK  KYPA--NVPL  TVDHDLYFTI  GVGIDPCA--  -T----CT--  368
Potri.001G248700  QNATPVATNF  TDALRSLNSI  KYPA--RVPL  KIDHSLFFTI  GLGVNPCA--  -T----CV--  371
Potri.009G042500  KNATPVATNF  TNALRSLNSI  KYPA--RVPL  KIDHSLFFTV  GLGVNPCA--  -T----CI--  370
Potri.016G112100  KNATAVANQF  TNSLRSLNSK  RFPA--KVPL  TVDHNLFFTV  GLGINPCP--  -T----CKAG  378
Potri.016G112000  KNATALANQF  TNSLRSLNSK  TFPA--KVPL  TVDHSLFFTV  GLGINPCP--  -T----CKAG  371
Potri.006G097000  KNATAVANQF  TNSLRSLNSR  RFPA--KVPL  NVDHNLFFTV  GLGVNPCP--  -S----CKAG  373
Potri.006G096900  KNATAVANQF  TNSLRSLNSR  RFPA--KVPL  NVDHNLFFTV  SLGVNPCP--  -S----CKAG  372
Potri.006G097100  KNATAVANQF  TNSLRSLNSR  RFPA--KVPL  NVDHNLFFTV  GLGVNPCP--  -S----CKAG  373
Potri.007G023300  SNDTEFALGY  NRKLRSLNTA  QFPA--NVPL  KVDRNLFYTV  GFGKDSCP--  -T----CV--  375
Potri.009G102700  PNDTAFARSY  NAKLRSLNSP  QFQA--NVPL  IVDRHLFYTI  GLGINPCP--  -T----CL--  374
Potri.004G156400  PNDTAFALSY  NAKLRSLNSP  QFPA--NVPL  KVDRHLFYTI  GLGINPCP--  -S----CL--  377
Potri.006G087500  FNDTTFATKF  VKKIRSLANA  RFPA--KVPK  KVDRRFFFTI  GLGSLPCSQN  KT----CQGP  392
Potri.009G156800  INATNVVANF  TRRLRSLANS  RFPV--NVPQ  TVDKKFFFTV  GLGNSPCPKN  QT----CQGP  389
Potri.009G156600  INATNVVANF  TRRLRSLANS  RFPV--NVPQ  TADKKFFFTV  GLGNSPCPKN  QT----CQGP  389
Potri.001G184300  INATNVVANF  TRKLRSLANS  RFPV--NVPQ  TVDKKFFFTV  GLGNSPCPKN  QT----CQGP  389
Potri.009G034500  INATNAVANF  TRKLRSLANF  QFPV--NVPQ  TVDKKFFFTV  GLGNNPCPKN  QT----CQGP  389
Potri.006G087100  INATGFVANF  TKKFRSLANA  KFPA--NVPQ  TVDRKFFFTV  GLGTNPCPKN  TT----CQGP  392
Potri.001G341600  LNDTAFAANF  TSKLRSLASA  QFPA--KVPQ  KVDMRFFFTV  GLGTNPCPKN  QT----CQGP  393
Potri.001G054600  LNDTSFATKF  TSKLRSLANA  QFPA--NVPQ  KVDRQFFFTV  GLGTHSCPQN  QT----CQGP  394
Potri.001G401300  LNDTSFATNF  TSKLRSLASA  EFPA--NVPQ  KVDRQFFFSV  SLGTNPCSKN  KT----CQGP  394
Potri.001G401100  LNDTSFATNF  TSKLRSLASA  EFPA--NVPQ  KVDRHFFFTV  GLGTNPCSKN  QT----CQGP  394
Potri.011G120300  LNDTSFATNF  SSKLRSLASA  DFPA--NVPQ  KVDRQFFFTV  GLGTNPCSKN  QT----CQGP  394
Potri.011G120200  LNDTSFATNF  SSKLRSLASA  DFPA--NVPQ  KVDRQFVFTV  GLGTNPCSKN  QT----CQGP  394
Potri.015G040700  MEDTAFATKF  SDKIRSLASS  QYPC--NVPK  TIDKRVITTI  SLNLQDCPEN  KT----CSGF  373
Potri.015G040800  MEDTAFATKF  SDKIKSLASP  QYPC--NVPK  TIDKRVITTI  SLNLQDCPEN  KT----CSGF  291
Potri.015G040600  MEDTAFATKF  SDKIKSLASP  QYPC--NVPK  TIDKRVITTI  SLNLQDCPEN  KT----CLGL  390
Potri.015G040400  MEDTAFATKF  SDKIKSLASP  QYPC--NVPK  TIDKRVITTI  SLNIQDCPEN  KT----CSGY  390
Potri.012G048900  MEDTAFATKF  SGNIKSLASP  KYPC--DVPK  TIDKRVITTI  SLNLQDCPAK  KT----CLGF  390
Potri.016G107900  FTDTPTAHKF  FTSITGLAGG  PHWV--PVPR  HIDEHMFVTV  GLGLSICPT-  ------CL--  378
Potri.016G106100  FTDTPTAHKF  FTSITGLAGG  PHWV--PVPR  HIDEHMFVTV  GLGLSICPT-  ------CL--  378
Potri.016G106300  FNDTPTAHKF  FTSITGLAGG  PHWV--PVPR  QIDEHMFVTV  GLGLSICPT-  ------CS--  371
Potri.016G107500  FTDTPTAHKF  FTSITGLAGG  PHWV--PVPR  QVDEHMFVTV  GLGLSICPT-  ------CL--  377
Potri.016G106000  FTDTPTAHKF  FTSITGLAGG  PHWV--PVPR  QVDEHMFVTV  GLGLSICPT-  ------CL--  377
Potri.006G094100  FNDTPTAHKF  FTNITGLAGG  PHWV--PVPR  QIDEHMFVTM  GLGLSICPT-  ------CS--  370
Potri.019G121700  FNDTNSAIAF  TSSLRS----  PSKV--NVPL  QIDENLFFTV  GFGLINCTNP  --NSPRCQGP  388
Potri.019G124300  FNDTNSAIAF  TSSLRS----  PSKV--NVPL  QIDENLFFTV  GFGLINCTNP  --NSPRCQGP  388
Potri.013G152700  FNDTNSAIAF  TSRLRS----  PSKV--KVPL  QIDENLFFTV  GLGLINCTNP  --NSPRCQGP  388
Potri.010G183600  YNDTATVTAF  SGSLRS----  PRKV--EVPT  DIDENLFFTI  GLGLNNCPKN  S-RARRCQGP  387
Potri.008G073700  YNDTATVTAF  SRSFRS----  PRKV--EVPT  DIDENLFFTI  GLGLNNCPKN  F-RARRCQGP  386
Potri.008G073800  YNDTATVTTF  TTSFKS----  ADKT--LVPT  DIDESLFFTI  GLGLNPCPSN  FNKSSQCQGP  394
Potri.010G183500  FNDTATVTAF  TSSFKS----  TDKT--FVPT  DIDESLFFTV  GLGLNPCPPN  FNKSSQCQGP  394
Potri.014G100600  FNDNLAVKTV  MDGLRGLNTS  D------VPK  EIDTNLFLTI  GMNVNKC-RS  KTPQQNCQGL  384
Potri.019G088900  FNDSAAAENY  TSRVRGLASR  DHPV--NVPQ  TINRRLYITI  ALNYLPC-TE  AT----CINS  381
Potri.019G088800  FNDTAAAENY  TSRVRGLASR  DHPV--NVPQ  TINRRLYIAI  ALNNLSC-TE  AT----CINS  381
Potri.019G088600  FNDTAAAENY  TSRVRGLASR  DHPV--NVPQ  TINRRLYIAI  ALNFLPC-TE  AT----CTGP  381
Potri.019G088700  FNDTAAAENY  TSRVRGLASR  DHPV--NVPQ  TVNRRLYITI  ALNRLPC-TE  AT----CTGP  381
Potri.019G088500  FNDTAAAENY  TSRVRGLASR  DHPV--NVPQ  IINRRLYITI  ALNFLPC-TE  AT----CNSS  381
Potri.001G206200  YNRTQASTDF  TKQFRS----  --PVKAHVPQ  KVDTRLFFTI  SVNLLNCSTD  KP----CAGP  370
Potri.005G200700  YDDTNAAYSF  LSSLRSLADE  DHPV--RVPS  NITTRIVSTL  SVNALPCHRN  RS----CEGP  379
Potri.011G071100  FNDTTALVNF  TGRLRSLADN  NHPI--HVPL  SISTTLFFTV  SVNRFTC-AN  TS----C-GA  373
Potri.005G200600  FNDTTASVNF  TGRLRSLADN  NHPI--YVPM  SISTPLFFTV  SVNIFTC-AN  TS----C-GA  377
Potri.005G200500  FNDTTASVNF  TGRLRSLADN  NHPI--HVPM  SISTPLFFTV  SVNIFTC-AN  TS----C-GA  377
```

Sequence logo

4.3bits

0.0bits

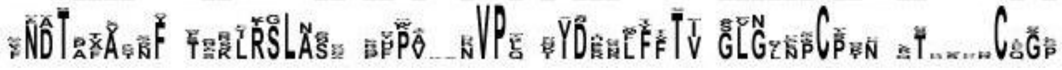

Supplementary Figure S1 (cont.)

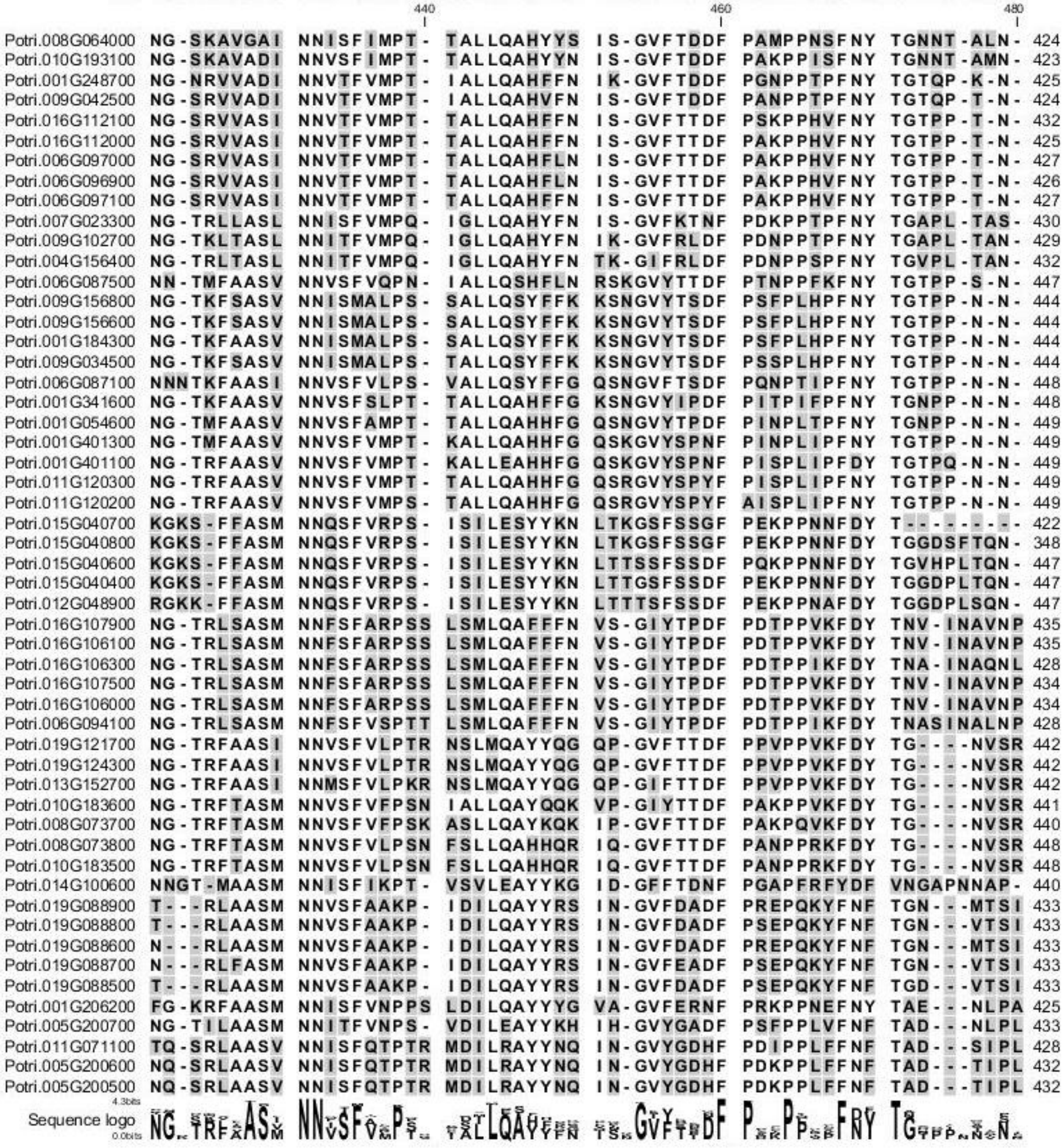

Supplementary Figure S1 (cont.)

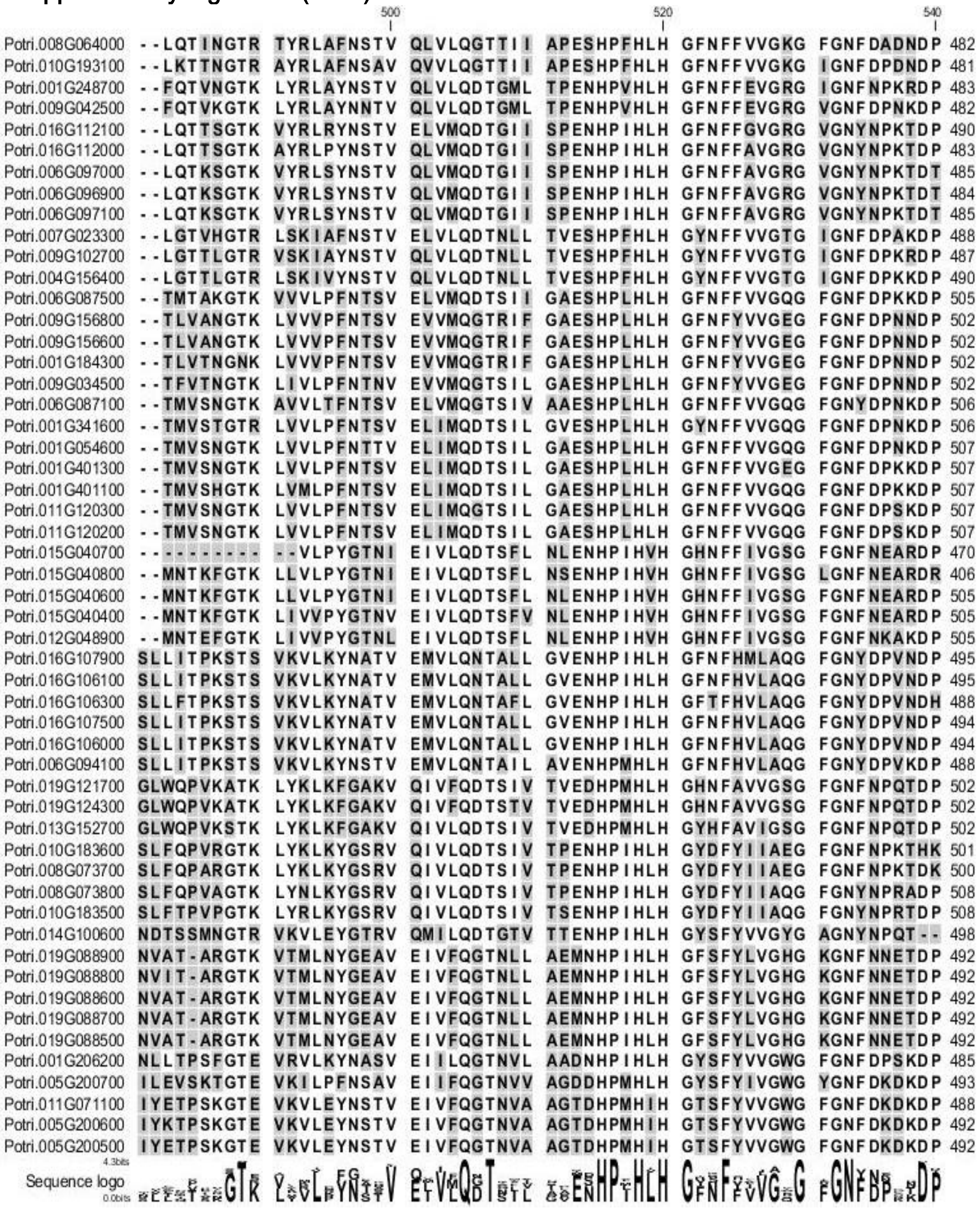

Supplementary Figure S1 (cont.)

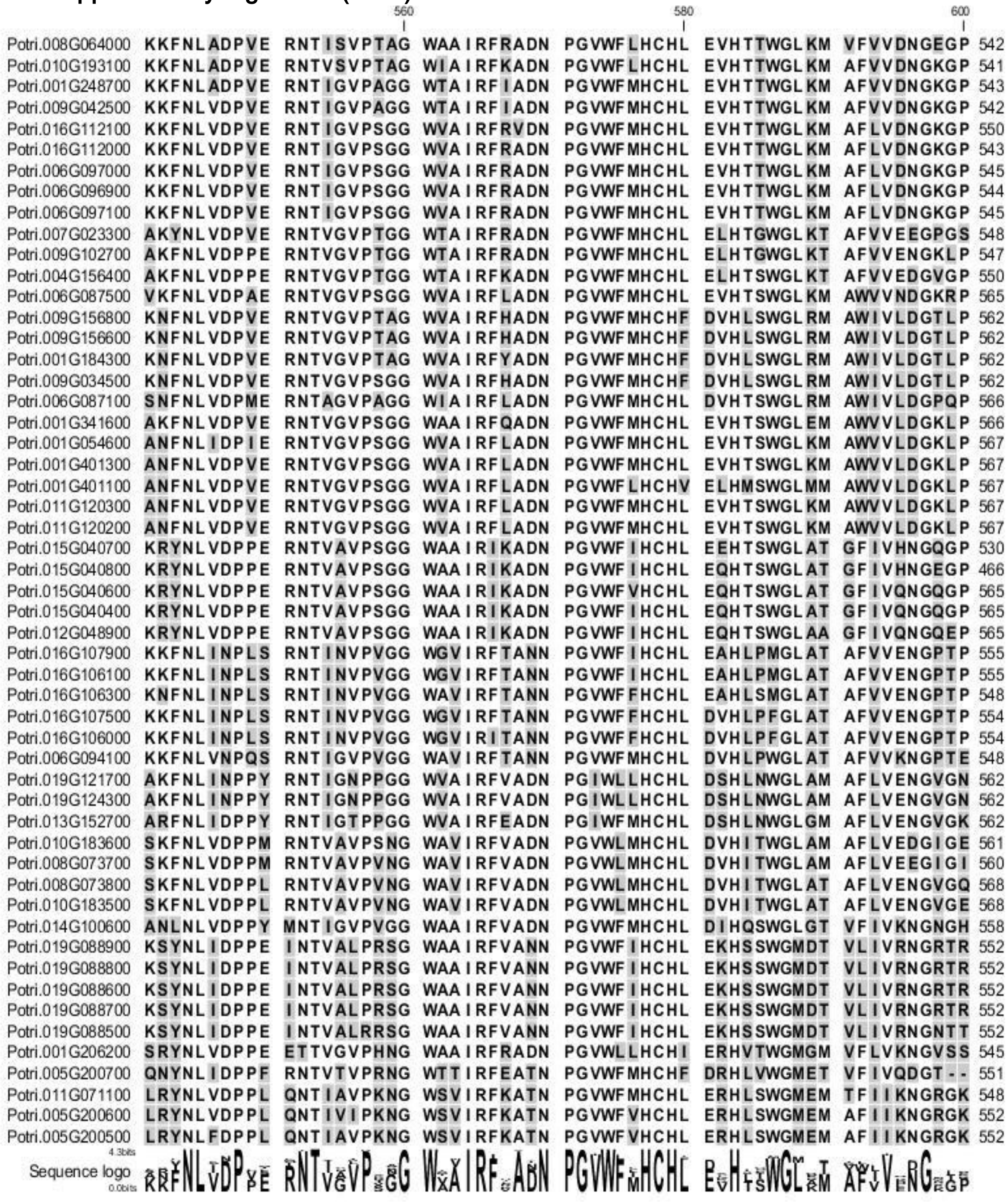

Supplementary Figure S1 (cont.)

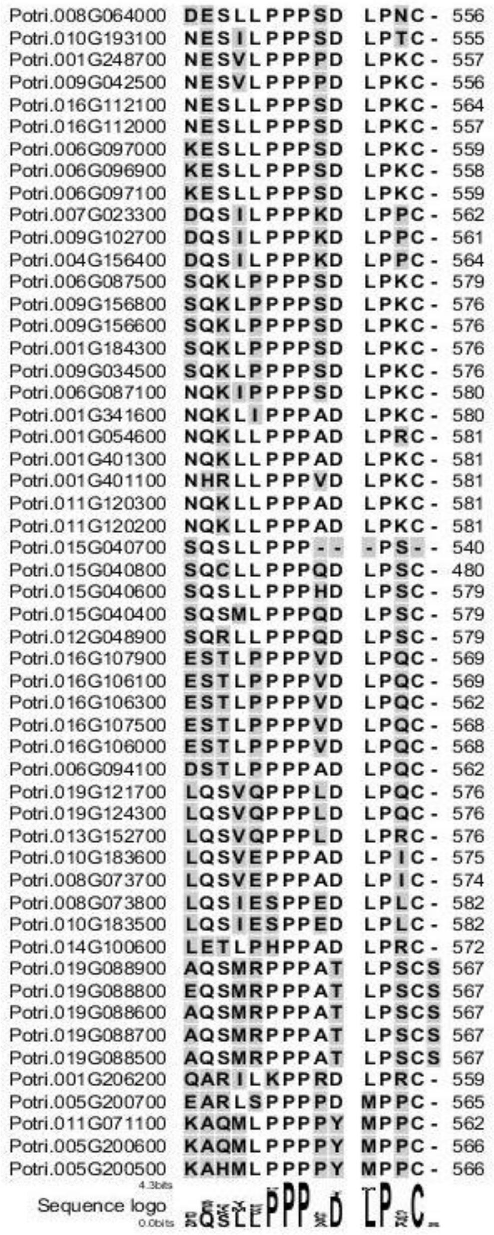

Figure S1. Amino acid alignment of *Populus trichocarpa* laccases. Alignment was created with CLC Main Workbench software (www.clcbio.com). Most common amino acid per position is noted with Sequence logo at base of alignment. Divergent residues are shaded in grey.

Supplementary Figure S2

```
                                                           20                                40                                60
                                                           |                                 |                                 |
Potri.008G064000             M---------  ----------  --------EN  YRARAILLLV  IFIFPALVEC  EVRLYDFRVV  33
Potri.010G193100             M---------  ----------  --------EY  YQAR-TMLLV  IFIFPALVEC  KVRLYNFRVV  32
cassava4.1_004807m.mes       M---------  ----------  -------ACS  LLPTTTLLLL  IFLFPTFVES  AIRHYNFTVV  34
Gorai.011G279600.1.gra       M---------  ----------  ----------  VCWVRTLLFI  SVLVPAFVEC  RIRHYHFNVV  31
Eucgr.G03028.1.egr           M---------  ----------  --RRSEGAEM  ERWFPAMLVV  AVMLPAAAEG  LIRHYKFSVV  39
Medtr5g081810.1.mtr          M---------  ----------  ----------  ----------  ----------  -----THNVV  6
PGSC0003DMP400033331.stu     M---------  ----------  ----------  ESWLRILFLF  ACLFPAFVEC  RIRRYNFNVM  31
AT2G38080                    M---------  ----------  -------GSH  MVWFLFLVSF  FSVFPAPSES  MVRHYKFNVV  34
LOC_Os11g48060.1.osa         MHCTALSPAL  SSPSPAAGHA  ANMAVLPESR  RLSLLLMAAC  FLLQALSAHA  ITRHYKFNVV  60
GRMZM2G072808_P01.zma        M---------  ----------  --ATPYRLPC  CCYALVTVLV  LFFSVDATEG  AIREYQFDVQ  39

                                                           80                               100                               120
                                                           |                                 |                                 |
Potri.008G064000             LTNTTKLCST  KSIVTINGKF  PGPTIYAREG  DNVNIKLTNH  VQYNVTIHWH  GVRQLRTGWS  93
Potri.010G193100             LTNTTKLCST  KSIPTINGKF  PGPTIYAREG  DNVNIRLTNQ  VQYNVTVHWH  GVRQLRTGWA  92
cassava4.1_004807m.mes       MKNTTKLCSS  KSIATINGKF  PGPTLYARED  DTVNVRVSNN  IQYNVTIHWH  GVRQLRTGWS  94
Gorai.011G279600.1.gra       VKNATKLCST  KPIVTVNGTF  PGPRLYAREG  DNVLVRLTNH  VQYNVTIHWH  GVRQLRTGWS  91
Eucgr.G03028.1.egr           MKNVTKLCAS  KPIATVNGKF  PGPTLYARED  DTVLVRVVNH  VSYNVTIHWH  GVRQLRTGWS  99
Medtr5g081810.1.mtr          LKNETKLCST  KSFVSVNGKF  PGPTLYARED  DTLIVRVTNL  VQHNVTIHWH  GIKQLRTCWS  66
PGSC0003DMP400033331.stu     MKTTTRLCSS  KPIATVNGKF  PGPTIYAREG  DNVLVNVVNH  VKYNVSIHWH  GVRQLRTGWS  91
AT2G38080                    MKNVTRLCSS  KPTVTVNGRY  PGPTIYARED  DTLLIKVVNH  VKYNVSIHWH  GVRQVRTGWA  94
LOC_Os11g48060.1.osa         MRNMTRLCST  KPILTVNGKF  PGPTLYAREG  DNVLVKVVNH  VAHNVTIHWH  GVRQIRTGWY  120
GRMZM2G072808_P01.zma        MTNVTRLCSS  KSIVTVNGQF  PGPTVFAREG  DFVVIRVVNH  VPYNMSIHWH  GIRQLRSGWA  99
                                                                                             CBR I
                                                          140                               160                               180
                                                           |                                 |                                 |
Potri.008G064000             DGPAYITQCP  IRPGQSYLYN  FTLTGQRGTL  LWHAHISWLR  ATIHGAIVIL  PQKGVPYPFP  153
Potri.010G193100             DGPAYITQCP  IQPGQSYLYN  FTLTGQRGTL  LWHAHISWLR  ATIHGAIVIF  PKKGVPYPFP  152
cassava4.1_004807m.mes       DGPAYITQCP  IQPGQSFLYN  FTLTGQRGTL  LWHAHISWLR  TTIHGAIVIF  PKKGVPYPFP  154
Gorai.011G279600.1.gra       DGPAYITQCP  IQPGQNFLYN  FTLTGQRGTL  LWHAHISWLR  TTVHGAIVIL  PKKGVPYPFP  151
Eucgr.G03028.1.egr           DGPAFITQCP  IQPGQSYIYN  FTLTGQRGTL  LWHAHITWLR  STLHGAIVIL  PKRGVPYPFP  159
Medtr5g081810.1.mtr          DGPAYVTQCP  IQTGQSFVYN  FTITGQRGTL  LWHAHITWLR  ATMHGAIVIL  PKRGTPYPFP  126
PGSC0003DMP400033331.stu     DGPAYITQCP  IQPGQNYVYN  FTITGQRGTL  FWHAHILWLR  ATMHGAIVIL  PKLGVPYPFP  151
AT2G38080                    DGPAYITQCP  IQPGQVYTYN  YTLTGQRGTL  WWHAHILWLR  ATVYGALVIL  PKRGVPYPFP  154
LOC_Os11g48060.1.osa         DGPAYITQCP  IQPGSSFLYN  FTITGQRGTL  LWHAHINWLR  ATVHGAIVIL  PKLGVPYPFP  180
GRMZM2G072808_P01.zma        DGPAYITQCP  IQSGQSYVYK  FTITGQRGTL  WWHAHISWLR  ATVYGPIVIL  PKPGVPYPFP  159
                                                                          CBR II
                                                          200                               220                               240
                                                           |                                 |                                 |
Potri.008G064000             KPDKEKIIIL  GEWWKADVEA  VVNQATQTGL  PPNISDAHIV  NGQTGAVPGC  P-SP-GFTLH  211
Potri.010G193100             KPDKEKIIIL  SEWWKADVEA  VVNQATMTGL  PPNISDAHTV  NGHTGAVPGC  T-SP-GFTLH  210
cassava4.1_004807m.mes       KPDKEKLIIL  SEWWKADTEA  VINQAMQTGL  PPNISDSHTI  NGHVGPATGC  T-SQ-GYTLH  212
Gorai.011G279600.1.gra       KPYKEKVIVL  GEWWKADTEA  VVKQATQTGL  PPNISDAHTI  NGHPGPVPNC  S-SDDAYTLH  210
Eucgr.G03028.1.egr           KPYKEKTIIF  GEWWKADTEL  VLSQSVQSGM  PPNKSDSHTI  NGYPGPLPNC  S-SQ-GYELQ  217
Medtr5g081810.1.mtr          KPDKEKIIIL  GEWWKSDVEA  VVNQATSSGM  PPNISDAHTI  NGHPGPVPGC  I-SQ-GYTLH  184
PGSC0003DMP400033331.stu     KPDHEAVVVL  AEWWKSDTEA  VINQAIKSGL  APNVSDAHTI  NGHPGAISNC  P-SQGGYTLS  210
AT2G38080                    KPDNEKVIVL  GEWWKSDTEN  IINEALKSGL  APNVSDSHMI  NGHPGPVRNC  P-SQG-YKLS  212
LOC_Os11g48060.1.osa         APHKEAVIVL  GEWWKEDTET  VINQAMQLGV  GPNISDAHTI  NGHPGPLSEC  ASSQDGFKLS  240
GRMZM2G072808_P01.zma        APYDEVPVLF  GEWWTADTEA  VISQALQTGG  GPNVSDAFTI  NGLPGPLYNC  S-AKDTFKLK  218

                                                          260                               280                               300
                                                           |                                 |                                 |
Potri.008G064000             VESGKTYLLR  IINAALNDEL  FFKIAGHNIT  VVEVDAAYTK  PFSTDTIFIG  PGQTTNALLT  271
Potri.010G193100             VESGKTYLLR  IINAALNDEL  FFKIAGHNIT  VVEVDATFTK  PFSTDTIFIG  PGQTTNALLT  270
cassava4.1_004807m.mes       VEPGKTYLIR  LVNAAINDEL  FFKIAGHNLK  IVEVDASYTK  PFTTDTIFIG  PGQTTNALLT  272
Gorai.011G279600.1.gra       VETGKTYLLR  VINAAVNDEL  FFKIANHNLT  VVEVDACYTK  PFETDTLFLG  PGQTTTALLK  270
Eucgr.G03028.1.egr           VESGKTYLLR  IVNAAVNDEL  FFKIAGHNLT  VVEVDASYTK  PFSIDTIFIA  PGQTTNALLA  277
Medtr5g081810.1.mtr          VESGKTYLLR  IINAALNDEL  FFKIAGHKLI  VVEADASYLK  PFEIDTIFLS  PGQTTNVLLT  244
PGSC0003DMP400033331.stu     VDPGKSYMLR  VINAALNEEL  FFKIAGHKMT  VVEVDATYVK  PFKTDTIIIA  PGQTTNVIVT  270
AT2G38080                    VENGKTYLLR  LVNAALNEEL  FFKVAGHIFT  VVEVDAVYVK  PFKTDTVLIA  PGQTTNVLLT  272
LOC_Os11g48060.1.osa         VENGKTYMLR  IINAALNDDL  FFKVAGHELT  VVEVDAVYTK  PFKTDTLLIT  PGQTTNVLVR  300
GRMZM2G072808_P01.zma        VKPGKTYMLR  IINAALNDEL  FFSIAGHPLT  VVDVDAVYIK  PITVETIIIT  PGQTTNVLLT  278

                                                          320                               340                               360
                                                           |                                 |                                 |
Potri.008G064000             ADKSV--GKY  LMAVSPFMDT  -VVAVDNVTA  IAFLRYKGTI  AFSPPVLTTT  ----------  318
Potri.010G193100             ADKSI--GKY  LIAVSPFMDT  -VVAVDNVTA  IAFLRYKGTL  AFSPPVLTTT  ----------  317
cassava4.1_004807m.mes       TDQAT--GKY  LIAVSPFMDT  -IVAVDNVTA  IAFLRYKDTL  AFSPPVLTST  ----------  319
Gorai.011G279600.1.gra       ADQGI--GKS  LIAISPFMDT  -TVAVNNLTG  IGYLRYNHTL  AFTPTTFVAI  ----------  317
Eucgr.G03028.1.egr           ADKSG--GSY  IMAISPFMDT  -VVAVDNLTA  TGIVQYKGTI  ASAPTVQASI  ----------  324
Medtr5g081810.1.mtr          ANQPI--GKY  LIAITPFMDA  -PIGFDNLSS  IATLRYKGIP  PYTKTILTNI  ----------  291
PGSC0003DMP400033331.stu     ANKGS--GKY  MVAASPFMDA  -PIAVDNVTA  TATLHYSGTL  ASSITTLTNT  ----------  317
AT2G38080                    ASKSA--GKY  LVTASPFMDA  -PIAVDNVTA  TATVHYSGTL  SSSPTILTLP  ----------  319
LOC_Os11g48060.1.osa         ANQGA--GRY  LLSVSPFMDA  -PVQVDNKTG  TATLHYANTV  SSSMASLTLV  K--------P  349
GRMZM2G072808_P01.zma        TKPSYPGATY  YMLAAPYSTA  RPGTFDNTTV  AGILEYEDPT  SSPPPHAAFD  KNLPALKPTL  338
```

Supplementary Figure S2 (cont.)

			380		400	420	
Potri.008G064000	PAINATPVTS	TFMDNLRSLN	SKKFPANVPL	TVDHSLYFTI	GVGIDPCAT-	--CVNG---S	372
Potri.010G193100	PAINATPATS	TFMDKLRSLN	SKKYPANVPL	TVDHDLYFTI	GVGIDPCAT-	--CTNG---S	371
cassava4.1_004807m.mes	PAINATAVTS	KFMDNLRSLN	SKKYPANVPL	TVDHYLYFTI	GVGINSCPT-	--CVNG---S	373
Gorai.011G279600.1.gra	PAVNATPVTS	VFSDSLRSLN	SKQYPANVPL	TIDHSLFFTI	GVGINPCAT-	--CFNG---S	371
Eucgr.G03028.1.egr	PAINATSFEF	SFINSLRSLN	SKQYPAKVPL	TVDHSLFITM	SAGVNPCST-	--CVNG---K	378
Medtr5g081810.1.mtr	PPLNATPITK	TFTDSLRSLN	SKTYPTRVSL	TIDHSLLFAI	TVGLNPCDT-	--CITD---N	345
PGSC0003DMP400033331.stu	PPKNATPVAN	NFIDSLRSLN	SKKYPAKVPK	KVDHSLFFTV	GLGVNPCSS-	--CKQGN-GS	373
AT2G38080	PPQNATSIAN	NFTNSLRSLN	SKKYPALVPT	TIDHHLFFTV	GLGLNACPT-	--CKAGN-GS	375
LOC_Os11g48060.1.osa	PPQNATHIVS	KFTDSLHSLN	SKEYPANVPQ	TVDHSLLLTV	GVGVNPCPS-	--CING---T	403
GRMZM2G072808_P01.zma	PQINDTSFVA	NYTARLRSLA	TAEYPADVPR	EVHRRFFFTV	GLGTHPCAVN	GTCQGPTNSS	398

		440		460		480	
Potri.008G064000	KAVGAINNIS	FIMPTTALLQ	AHYYS-ISGV	FTDDFPAMPP	NSFNYTGNNT	ALNLQTINGT	431
Potri.010G193100	KAVADINNVS	FIMPTTALLQ	AHYYN-ISGV	FTDDFPAKPP	ISFNYTGNNT	AMNLKTTNGT	430
cassava4.1_004807m.mes	KSVGNINNVS	FVMPSTALLQ	AHYYN-ISGI	FTDDFPANPL	VPFNYTGNFT	GG-ISTMNGT	431
Gorai.011G279600.1.gra	RAVAAINNVS	FVMPTTAILQ	AHYYG-INGV	FTDDFPAKPA	IPFNYTGTPP	SG-VQTMNGT	429
Eucgr.G03028.1.egr	KLVAALNNVS	FVMPSTDILE	AHYYK-IKGV	YTDDFPGNPP	TPFNYTGTPP	SN-MQTTNGT	436
Medtr5g081810.1.mtr	KLVSAINNIT	FLMPTVSLLQ	ANYYN-IKGV	FTDDFPSKPP	MVFDYTGTDQ	PANLHTDNGT	404
PGSC0003DMP400033331.stu	RVVASINNVT	FVMPTVAILQ	AHFFG-IKGV	YTTDFPQNPP	FKFNYTGTPP	TN-LATMSGT	431
AT2G38080	RVVASINNVT	FIMPKTALLP	AHYFN-TSGV	FTTDFPKNPP	HVFNYSGGSV	TN-MATETGT	433
LOC_Os11g48060.1.osa	RVVGTINNVT	FIMPSTPILQ	AHYYN-IPGV	FTEDFPATPL	HKFNYTGSGP	KN-LQTMNGT	461
GRMZM2G072808_P01.zma	RFAASVNNVS	FVLPTTALLQ	SHFAGKSRGV	YSSNFPAAPL	VPFNYTGTPP	NN-TNVSNGT	457

		500		520		540	
Potri.008G064000	RTYRLAFNST	VQLVLQGTTI	IAPESHPFHL	HGFNFFVVGK	GFGNFDADND	PKKFNLADPV	491
Potri.010G193100	RAYRLAFNSA	VQVVLQGTTI	IAPESHPFHL	HGFNFFVVGK	GIGNFDPDND	PKKFNLADPV	490
cassava4.1_004807m.mes	RLFRIAFNST	VQIVLQGTGI	IAPESHPIHL	HGFNFFAIAK	GVGNFDLVND	PKKFNLVDPV	491
Gorai.011G279600.1.gra	KVYRLAYNST	VQLVIQGNTI	IAPESHPTHL	HGSNFFVVGR	GVGNFDPEKD	PLKFNLVDPV	489
Eucgr.G03028.1.egr	RVYKLAYNST	VQVVLQGTSI	IAPENHPTHL	HGFNFFGIGK	GLGNFDPNKD	PKNFNLVDPV	496
Medtr5g081810.1.mtr	KVYRLNFNSS	VQIVLQGTAM	IAPENHPFHL	HGFNFFVVGQ	GLGNFDPEKD	PLRFNLVDPI	464
PGSC0003DMP400033331.stu	KVYLLPYNAT	VQLVLQDTGI	ISPENHPIHL	HGFNFFAVGK	GIGNFNPKTD	PNNFNLIDPV	491
AT2G38080	RLYKLPYNAT	VQLVLQDTGV	IAPENHPVHL	HGFNFFEVGR	GLGNFNSTKD	PKNFNLVDPV	493
LOC_Os11g48060.1.osa	RVYRLPYNAS	VQVVLQDTGI	ISPESHPIHL	HGFNFFVVGK	GVGNYNPRTS	PSTFNLIDPI	521
GRMZM2G072808_P01.zma	KLVVLPYGTS	VELVMQGTSI	LGAESHPLHL	HGFNFFVVGQ	GFGNFDPAKD	PAKYNLVDPV	517

CBR III

		560		580		600	
Potri.008G064000	ERNTISVPTA	GWAAIRFRAD	NPGVWFLHCH	LEVHTTWGLK	MVFVVDNGEG	PDESLLPPPS	551
Potri.010G193100	ERNTVSVPTA	GWIAIRFKAD	NPGVWFLHCH	LEVHTTWGLK	MAFVVDNGKG	PNESILPPPS	550
cassava4.1_004807m.mes	ERNTISVPTG	GWTAIRFRAD	NPGVWFLHCH	LEVHTSWGLK	MAFVVDNGKG	PNESLPPPPS	551
Gorai.011G279600.1.gra	ERNTVSVPTA	GWTAIRFRAD	NPGVWFFHCH	LEVHTTWGLK	MAFLVENGRG	PNESIEPPPS	549
Eucgr.G03028.1.egr	ERNTIGVPTA	GWTAIRFRAD	NPGVWFLHCH	LEVHTTWGLK	MVFIVEDGDG	PNESLLPPPA	556
Medtr5g081810.1.mtr	ERNTLSVPNN	GWIAIRFRAD	NPGVWFLHCH	LEVHTTWGLK	MAFIVDNGRG	PSESILPPPK	524
PGSC0003DMP400033331.stu	ERNTIGVPSG	GWVAIRFRAD	NPGVWFMHCH	LEIHTTWGLK	MAFLVDNGKG	PNESLLPPPK	551
AT2G38080	ERNTIGVPSG	GWVVIRFRAD	NPGVWFMHCH	LEVHTTWGLK	MAFLVENGKG	PNQSILPPPK	553
LOC_Os11g48060.1.osa	ERNTIGVPTG	GWTAIRFRSD	NPGVWFMHCH	FEVHTSWGLK	MAFVVDNGKR	PSETLIPPPK	581
GRMZM2G072808_P01.zma	ERNTVGVPAA	GWVAIRFRAD	NPGVWFMHCH	LEVHVSWGLK	MAWLVLDGER	PNEKLLPPPS	577

CBR IV

Potri.008G064000	DLPNC*	557
Potri.010G193100	DLPTC*	556
cassava4.1_004807m.mes	DLPQC*	557
Gorai.011G279600.1.gra	DLPKC*	555
Eucgr.G03028.1.egr	DLPKC*	562
Medtr5g081810.1.mtr	DLPIC*	530
PGSC0003DMP400033331.stu	DLPKC*	557
AT2G38080	DLPKC*	559
LOC_Os11g48060.1.osa	DLPQC*	587
GRMZM2G072808_P01.zma	DLPTC*	583

Figure S2. Amino acid alignment of homologous laccases to *Pt*LAC2 (Potri.008G064000) across different plant species. Homologs were determined based on BLAST search through Phytozome v 10.3 (www.phytozome.jgi.doe.gov). Alignment was created using CLC Main Workbench software. Copper Binding Regions (CBR) are indicated with solid line below residues. Diverged residues are shaded grey.

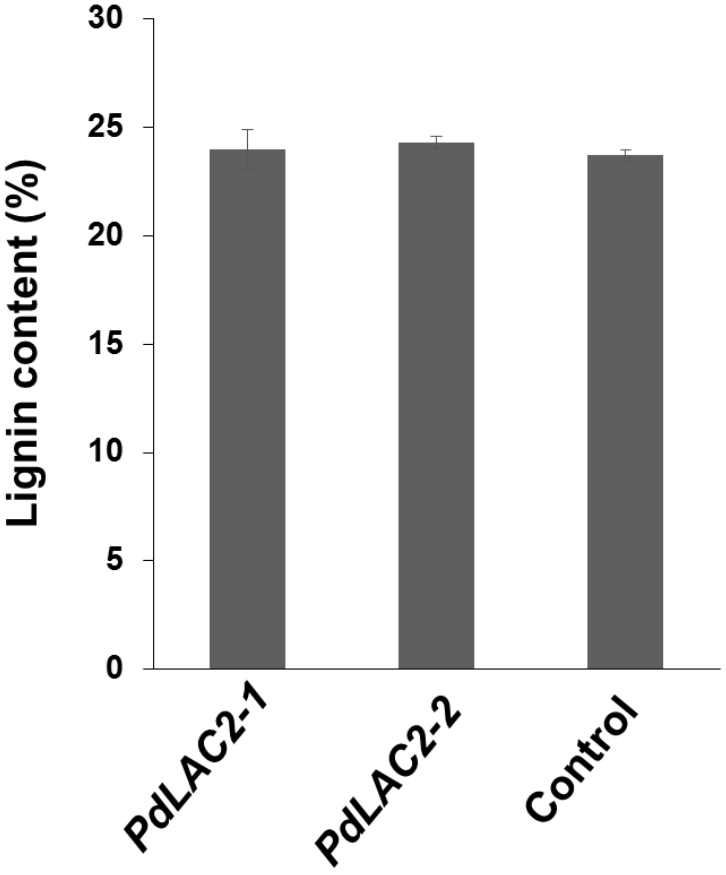

Figure S3. Lignin content in *PdLAC2* RNAi transgenic lines. Lignin content was measured through Molecular Beam Mass Spectrometry.

Supplemental Figure S4

Carbohydrate	Control	SD	*PdLAC2-1*	SD	*PdLAC2-2*	SD
Arabinose (Ara)	2.28	0.36	3.27	0.70	2.67	0.68
Rhamnose (Rha)	1.57	0.2	1.67	0.29	1.63	0.45
Fucose (Fuc)	0.17	0.06	0.15	0.07	0.20	0.00
Xylose (Xyl)	62.23	5.64	57.70	5.05	60.73	11.71
Galacturonic acid (GalA)	2.75	0.76	3.53	0.45	3.43	0.60
Mannose (Man)	1.92	0.13	2.27	0.57	2.27	0.46
Galactose (Gal)	1.68	0.29	1.57	0.21	1.83	0.57
Glucose (Glc)	27.42	7.13	29.90	7.19	27.20	13.50

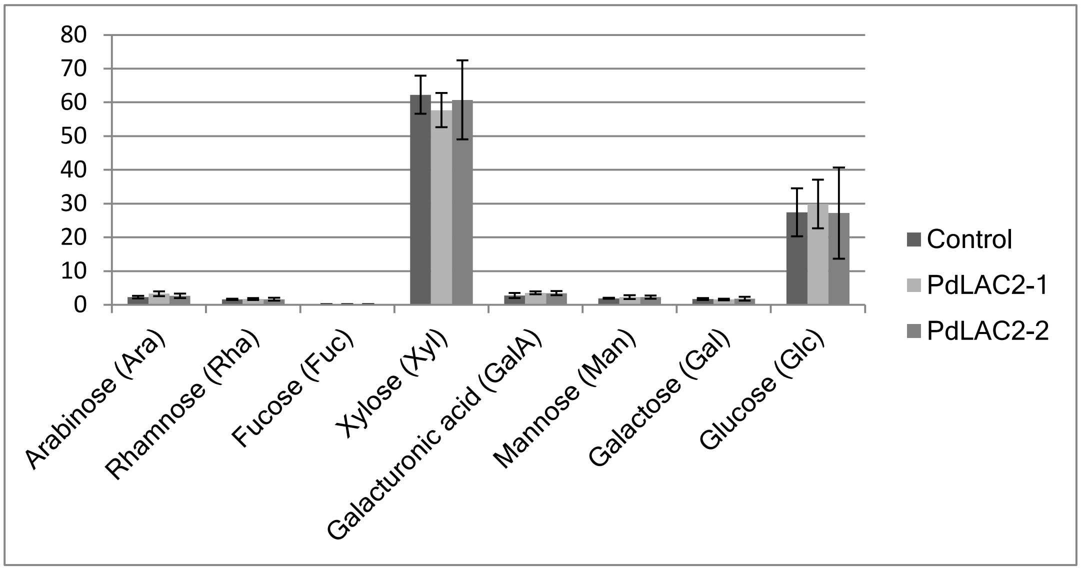

Figure S4. Carbohydrate analysis of *Pd*LACs transgenic lines and transgenic controls. Quantification is represented as percentage of each carbohydrate per sample.
